# Supplementary material for: Cropland expansion in the United States produces marginal yields at high costs to wildlife
Source: Nat Commun. 2020 Sep 9;11:4295. doi: 10.1038/s41467-020-18045-z (PMC7481238; doi:10.1038/s41467-020-18045-z)
Supplement: Supplementary file 1 — Supplementary Information [file 41467_2020_18045_MOESM1_ESM.pdf]

Supplementary Information for

Cropland expansion in the United States produces marginal yields at high costs to wildlife

Lark et al.

This file includes:

Supplementary Figures 1 – 17; 18

Supplementary Tables 1 – 10; 11

Supplementary Notes 1 – 3

Supplementary Methods

Supplementary References

Supplementary Figures

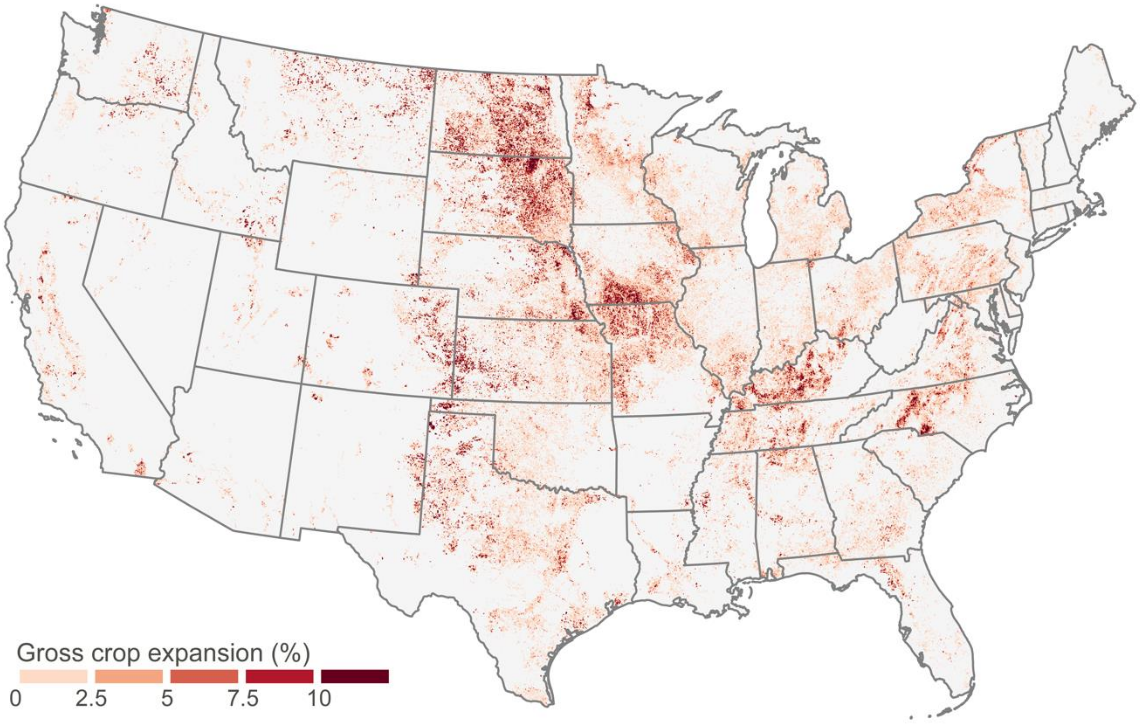

Supplementary Figure 1: Gross cropland expansion, 2008-16. The map represents the conversion of noncropland to cropland across the US, displayed as the percentage of the landscape that was converted between 2008-16. The highest rates of gross conversion occurred in the Prairie Pothole Region (PPR) of North and South Dakota, the Dissected Till Plains of Iowa and Missouri, and the High Plains portion of Kansas, Oklahoma, and Texas.

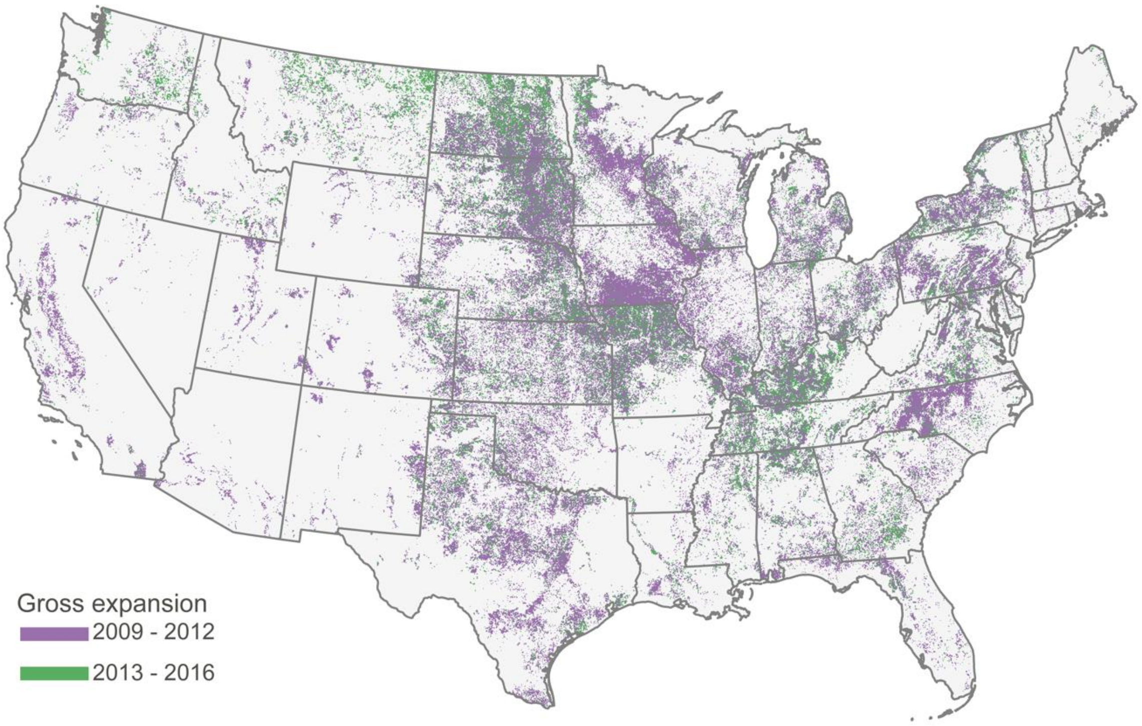

Supplementary Figure 2: Emerging hotspots of cropland expansion. The map displays areas of gross cropland expansion during 2009-12 compared to more recent areas of cropland expansion 2013-16. Midcontinental locations such as Kentucky, Missouri, and Tennessee, as well as the Canadian border in Montana and North Dakota have more recently emerged as additional hotbeds of elevated conversion.

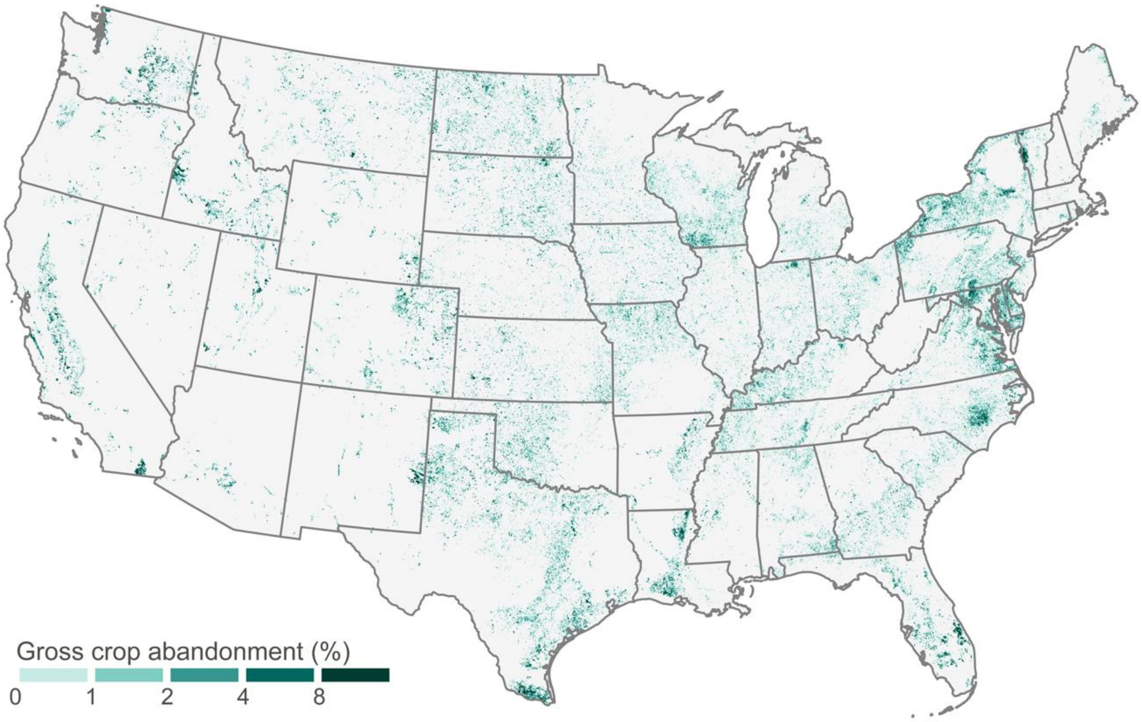

Supplementary Figure 3: Gross cropland abandonment, 2008-16. The map represents the conversion of cropland to noncropland across the US, displayed as the percentage of the landscape that was abandoned between 2008-16. Rates of cropland abandonment were greatest along the eastern seaboard, the Gulf coast, and parts of the Pacific Northwest.

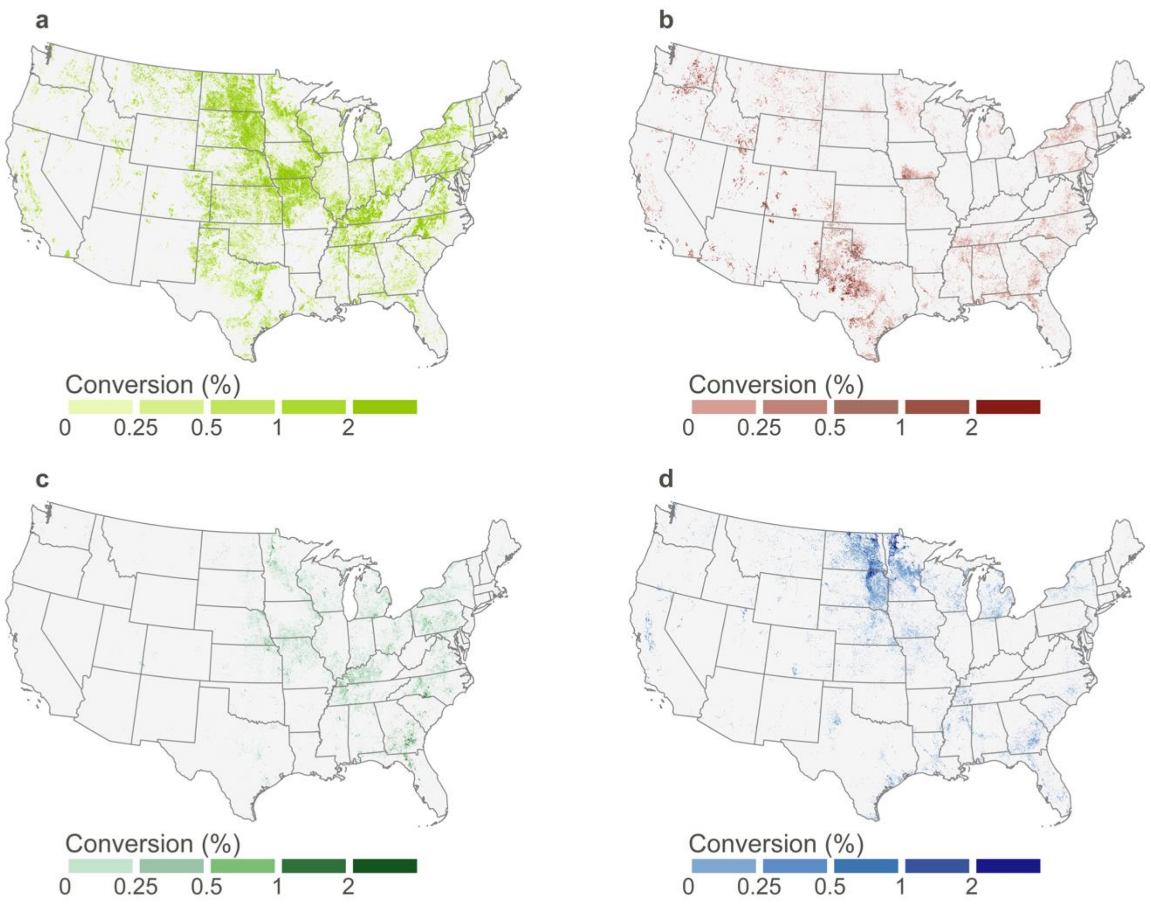

Supplementary Figure 4: Locations of conversion to cropland from specific land cover classes. The map displays the percentage of the landscape within 3 km x 3 km visualization units that has been converted to cropland from grasslands (**a**), shrublands (**b**), forests (**c**), and wetlands (**d**) between 2008-16. Land cover type derived from the Cropland Data Layer[1] based on the trajectory analysis of conversion and the latest non-crop class prior to a conversion. Grasslands were the primary source of new croplands across much of the country, including the Great Plains, the Midwest, and eastern states. Other regional patterns included the clearing of shrublands in in the western US, the conversion of forest and timber land in the southeastern US, and the cultivation of wetlands across the Prairie Pothole Region (PPR).

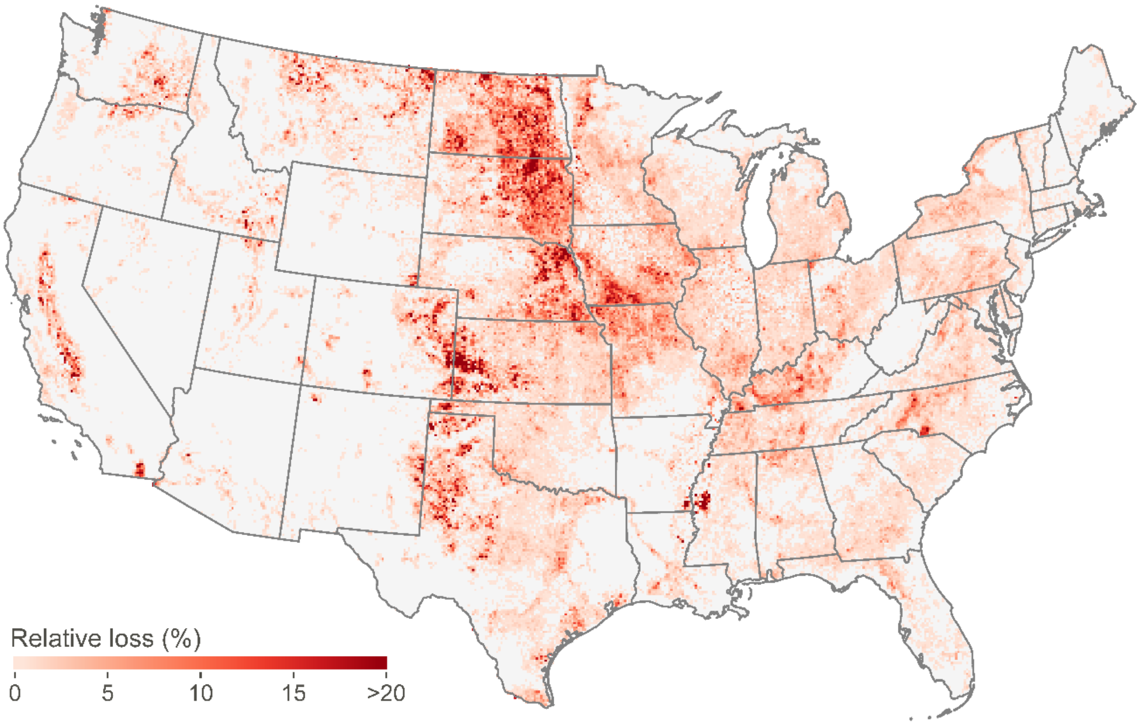

Supplementary Figure 5: Relative loss of all natural land covers combined. This map displays the conversion to cropland divided by the total amount of grasslands, shrublands, wetlands, and forest that were present in 2008. The ratio was assessed within nonoverlapping 9 km x 9 km neighborhoods. The highest rates of natural land cover loss relative to their remaining extent occurred in swaths of the Western Corn Belt and Southern Plains where rates of existing cultivation and cropland expansion were both high.

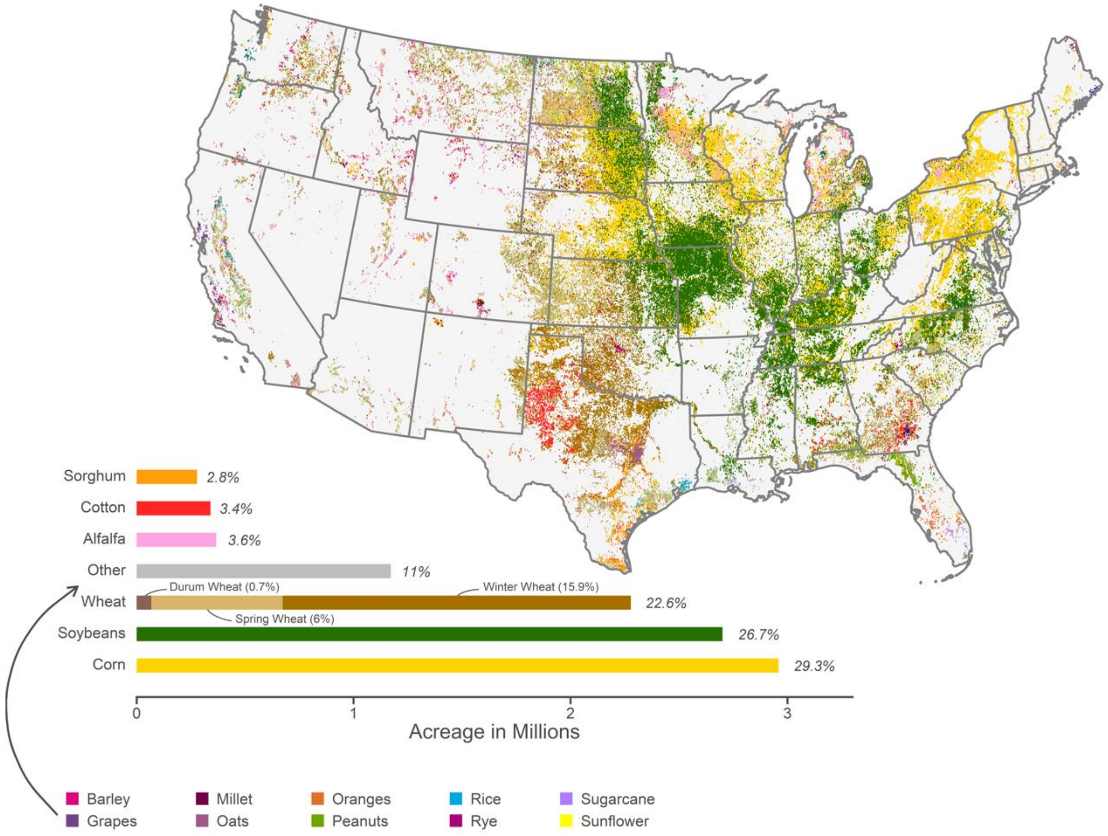

Supplementary Figure 6: First crop type planted on newly cultivated land. For each area of land converted to crop production, the first crop type was extracted from the Cropland Data Layer[1] for the first growing season following conversion. Between 2008-16, corn was the most common crop on land newly converted to cropland, followed by soybeans and wheat. Corn and soybeans were common throughout the midwestern Corn Belt and its periphery, whereas wheat was more common farther west.

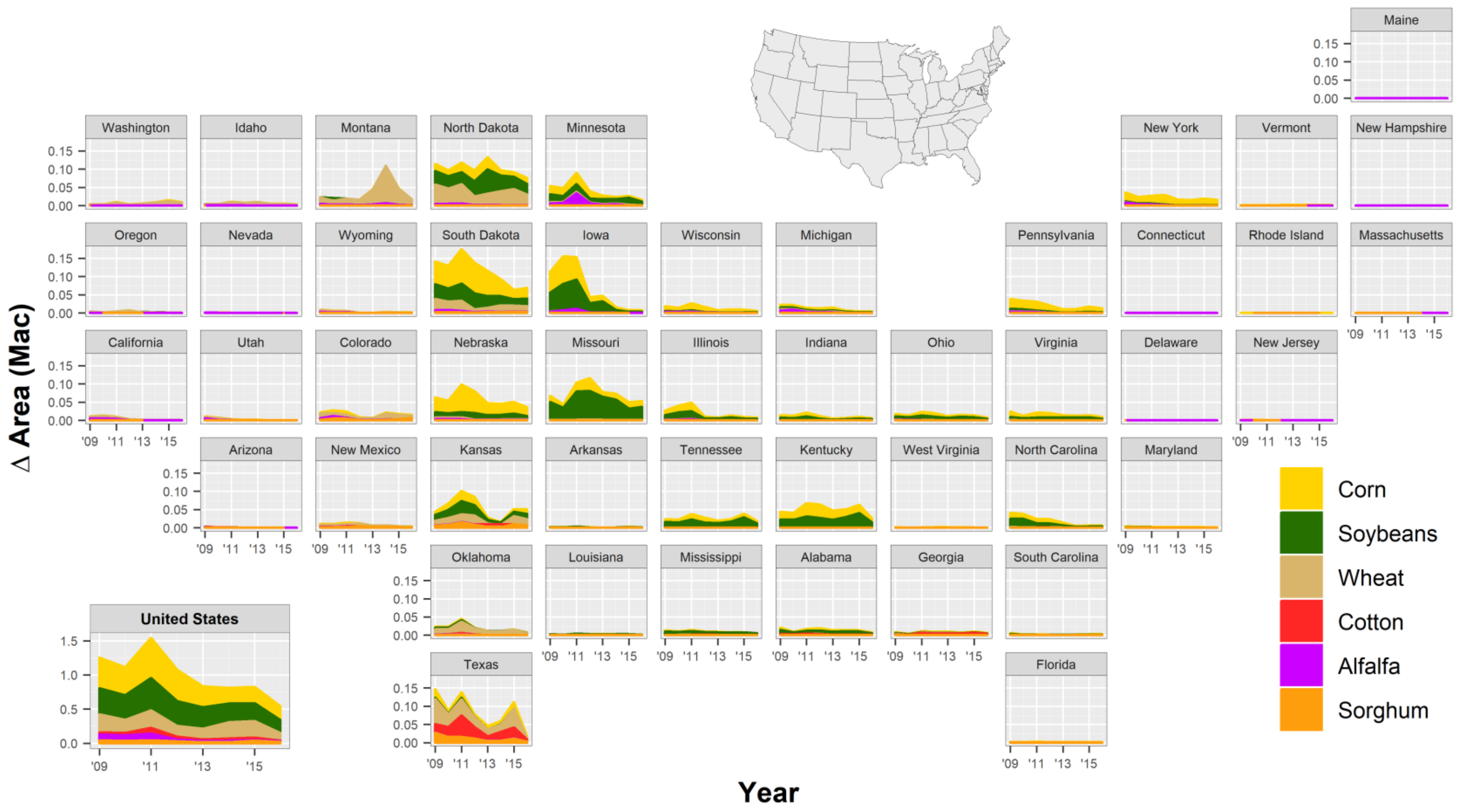

Supplementary Figure 7: First crop type planted on newly cultivated land by state and year. For each area of land converted to crop production, the first crop type was extracted from the Cropland Data Layer[1] for the first growing season following a conversion — i.e., land converted between 2008-09 is reported as a 2009 conversion to the crop present in 2009. Corn and soybeans were the most common on converted land in most states and years, with nearly equal proportions of each in many regions. In contrast, corn was much more common on new croplands in South Dakota, Nebraska, and New York, while soybeans were the more common breakout crop in Missouri and North Dakota. Nationwide across the United States, corn was most common on new croplands in all years except 2014-15 when soybeans were more prevalent.

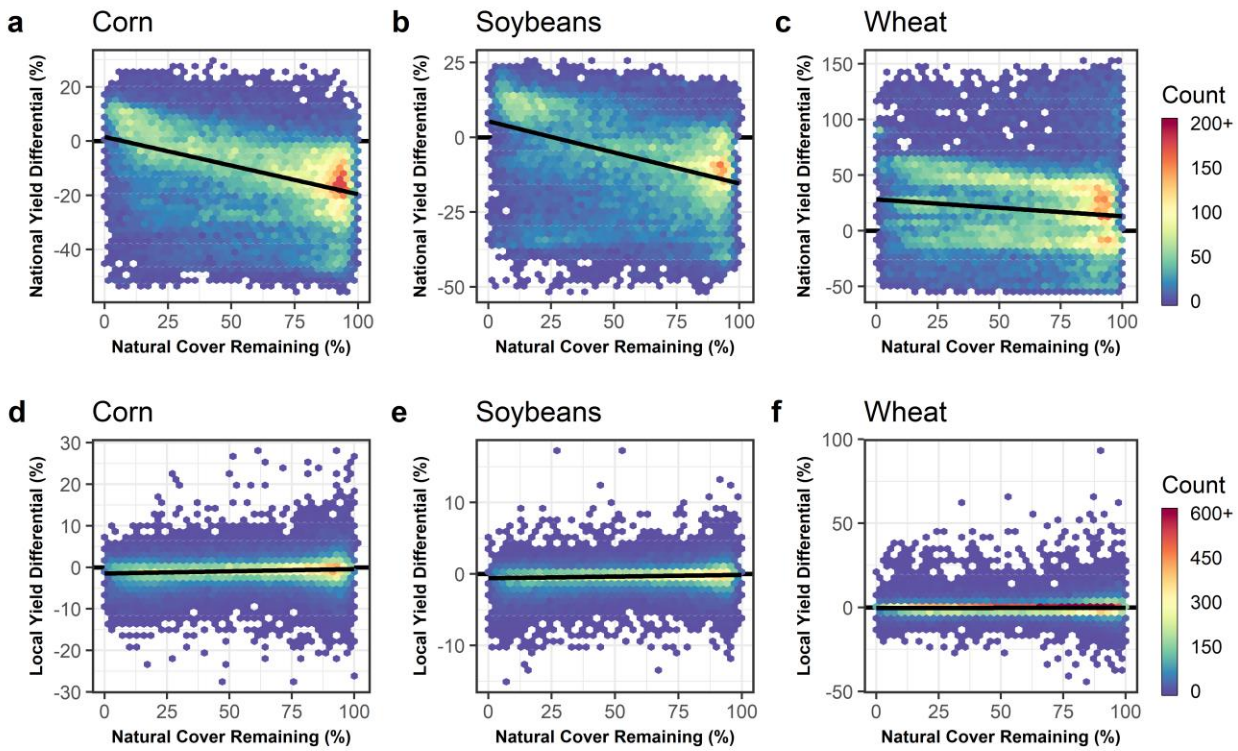

Supplementary Figure 8: Relationship between yield differentials and remaining uncultivated extent. In areas with more remaining natural land cover, new croplands have lower yields compared to national averages (a-c) but smaller local yield deficits compared to nearby croplands (d-f). In locations with little natural cover remaining, new croplands perform better in relation to national averages, but worse relative to nearby existing cropland extent. Trendlines represent the results of 0.5 (median) quantile regression. The slope of all six regressions was significantly different from zeros at $\alpha = 0.001$ (see **Supplementary Table 3** for details).

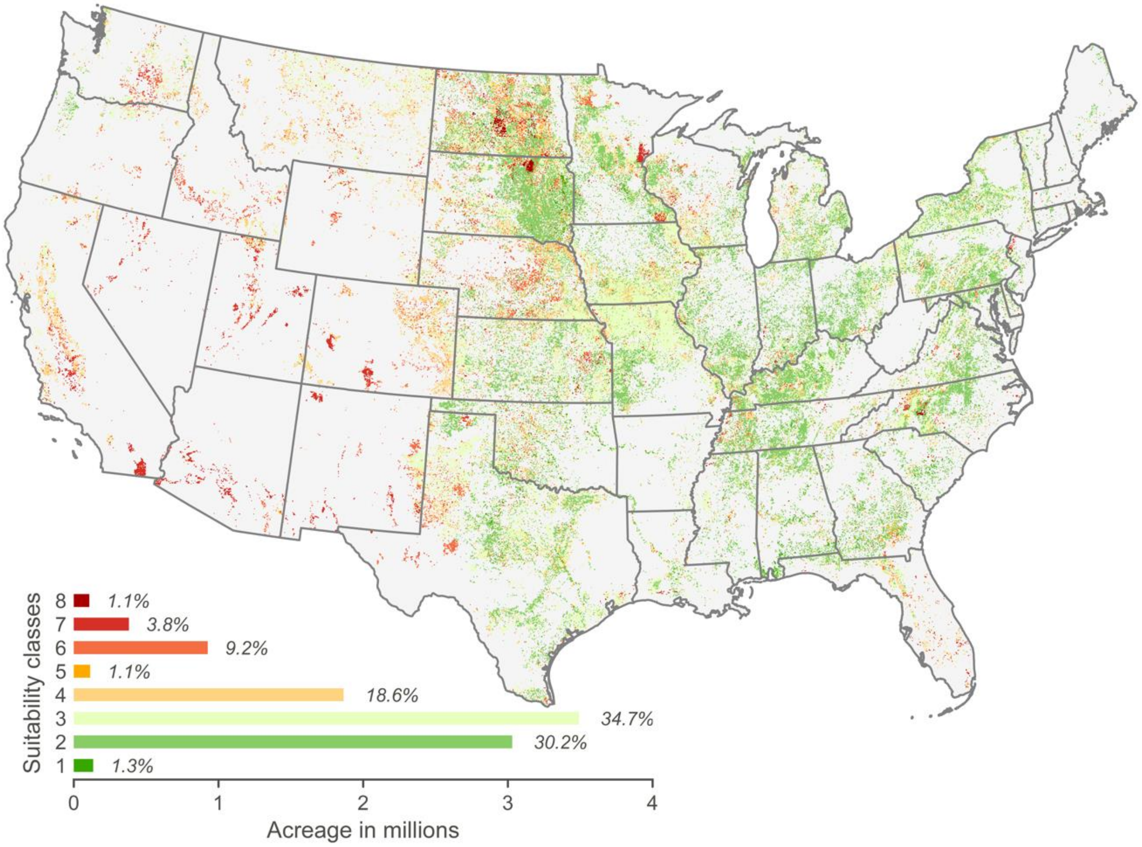

Supplementary Figure 9: Cultivation suitability of areas converted to cropland. Ratings are according to the USDA NRCS Land Capability Classes (LCC). Nearly two-thirds of converted lands were characterized as having moderate to severe limitations for cultivation (LCC 2-3). An additional 18% of conversion occurred on land with very severe limitations (LCC 4), and 15% occurred in areas deemed unsuitable for crop cultivation due to physical or environmental conditions that typically preclude tillage (LCC 5-8). In contrast, just 11% and 8.4% of existing croplands were considered to have very severe limitations or be unsuitable for cultivation, respectively (see **Supplementary Table 5**).

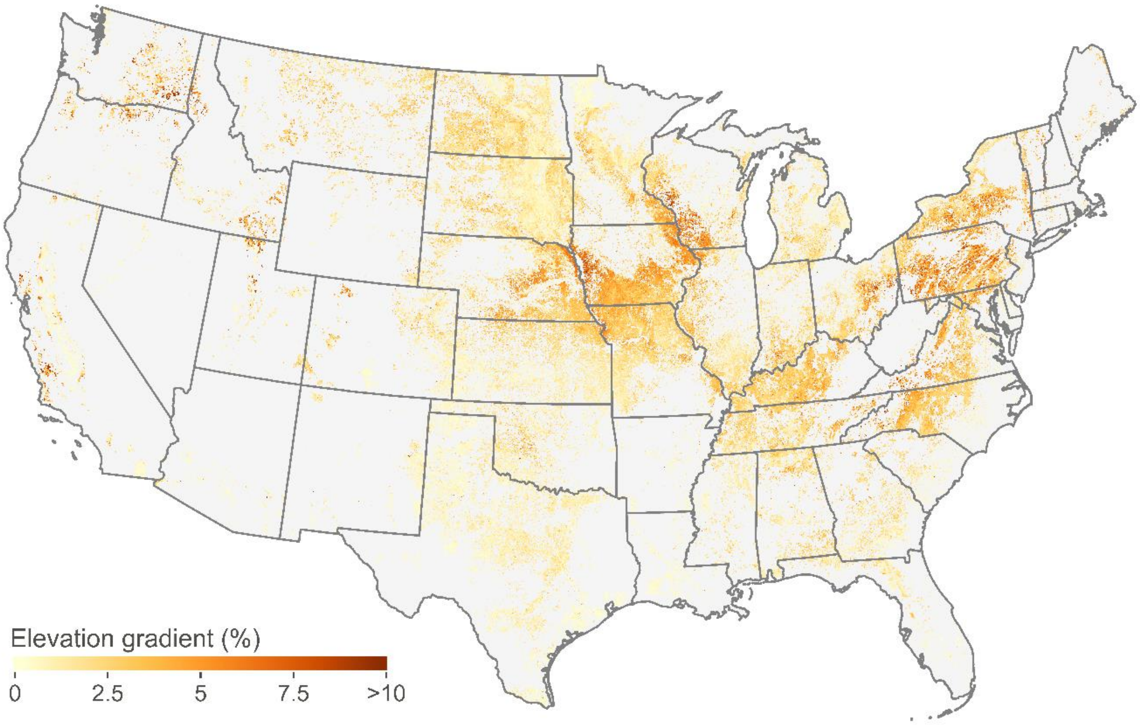

Supplementary Figure 10: Slope gradient of areas converted to cropland. Land recently converted to cropland had an average slope gradient of 3.35% (SD 3.45%), or approximately 1.7 times greater than that of existing croplands (mean 2.00%, SD 2.60%). Areas with particularly steep land converted to cropland included the periphery of the Appalachian Mountains, the Driftless region of southwestern Wisconsin and Iowa, and the Palouse Hills of Washington state.

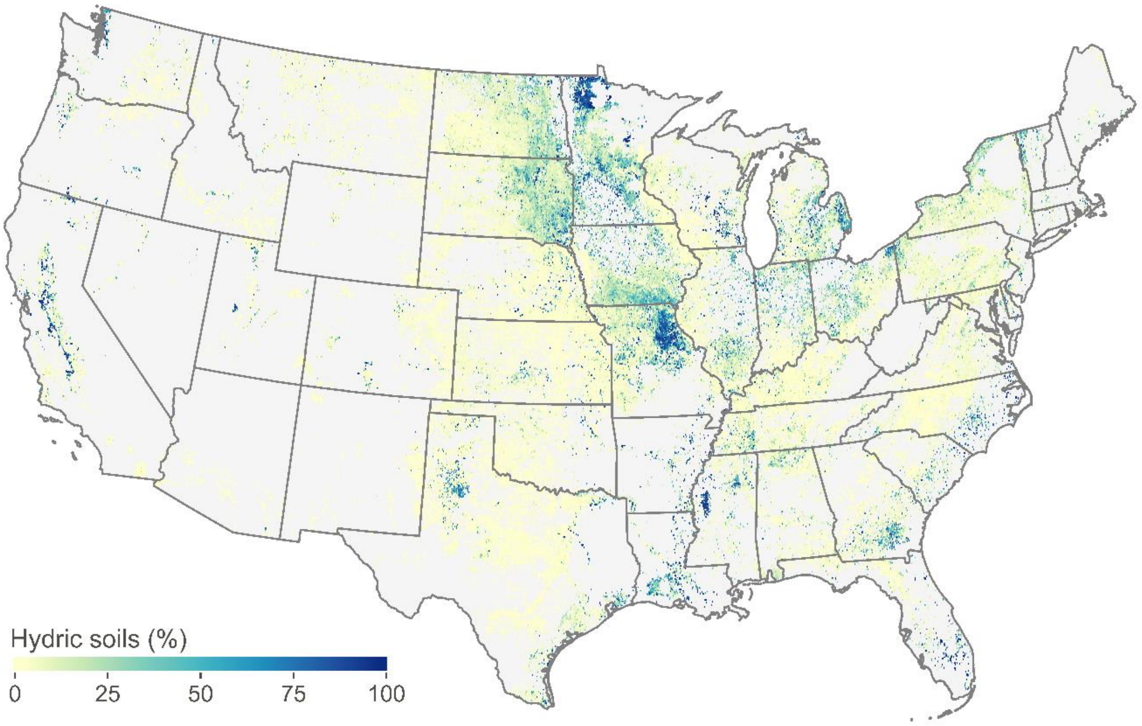

Supplementary Figure 11: Proportion of new croplands on hydric soils. On average, 8.10% of new croplands 2008-16 were planted on hydric soils, or those for which the topsoil is water saturated for at least part of the year. These wetland-capable locations can be highly productive, but also indicate likely use of drain tile, drainage ditches, or other wetness mitigation practices to facilitate crop production or expansion. Expansion occurred frequently on hydric soils throughout much of the Midwest region, especially in locations such as northern Minnesota and northeast Missouri, as well as in parts of the Southeast Coast and the Mississippi Alluvial Plain.

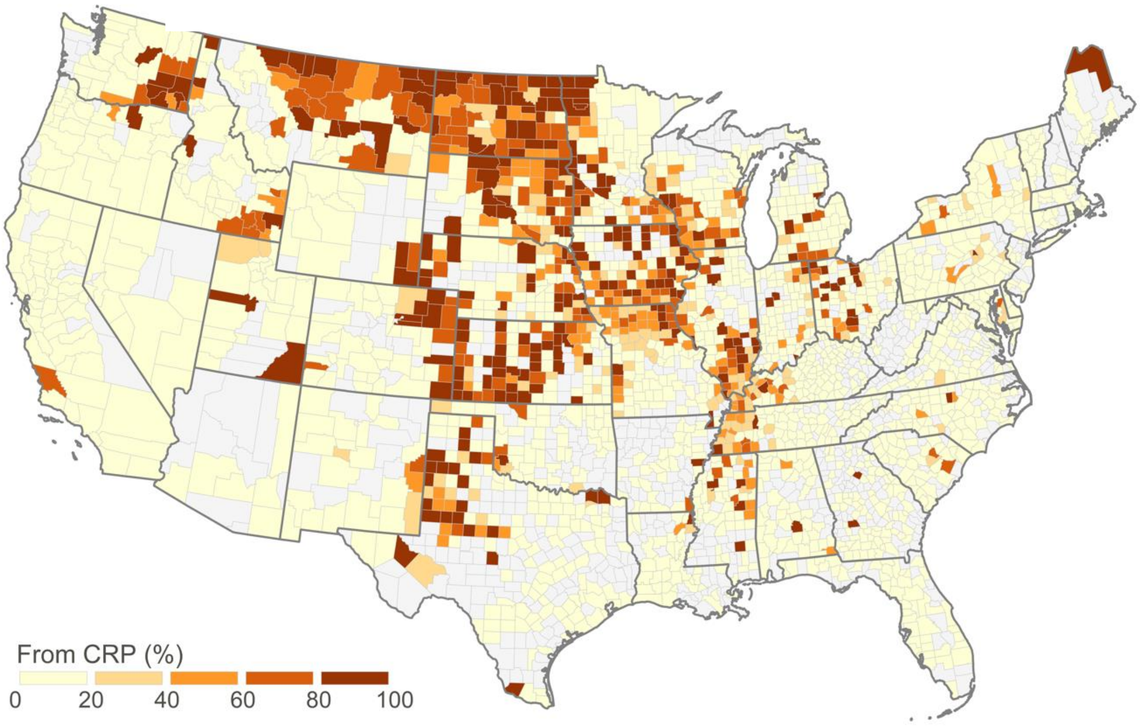

Supplementary Figure 12: Proportion of cropland expansion occurring on land previously enrolled in the Conservation Reserve Program (CRP). The map displays the estimated percentage of conversion to cropland coming from CRP within each county, derived from National Resources Inventory data for 2008-15. See **Supplementary Note 1** for details on the estimation and use of CRP conversion rates.

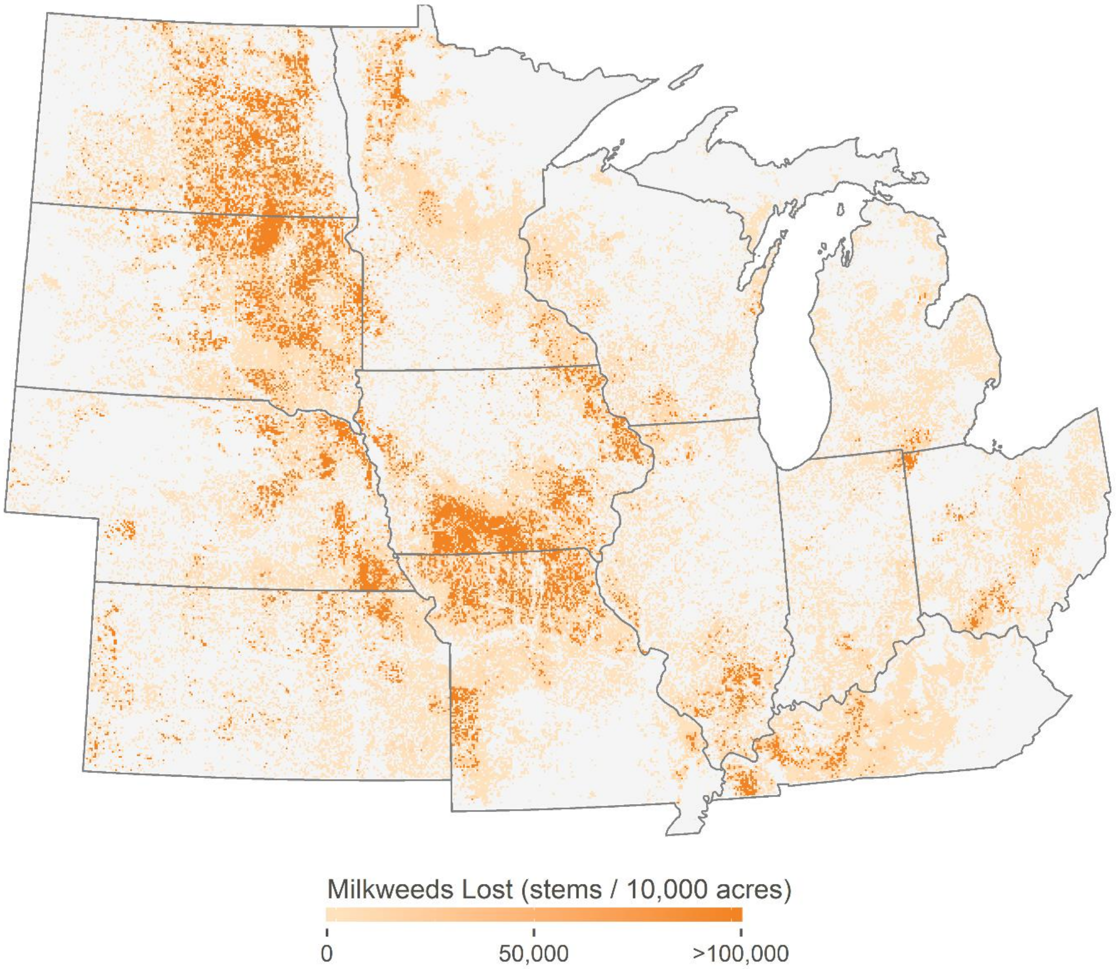

Supplementary Figure 13: Milkweed stems lost due to conversion of grasslands, shrublands, and wetlands to corn and soy production in the Midwest, 2008-16. The map represents the concentration of milkweed loss estimated as the number of stems lost per 10,000 acres of land in the region. The greatest losses of milkweed occurred in the eastern Dakotas, southern Iowa, and northern Missouri — locations with a confluence of high rates of cropland expansion and a high proportion of conversion from land previously enrolled in the Conservation Reserve Program (CRP).

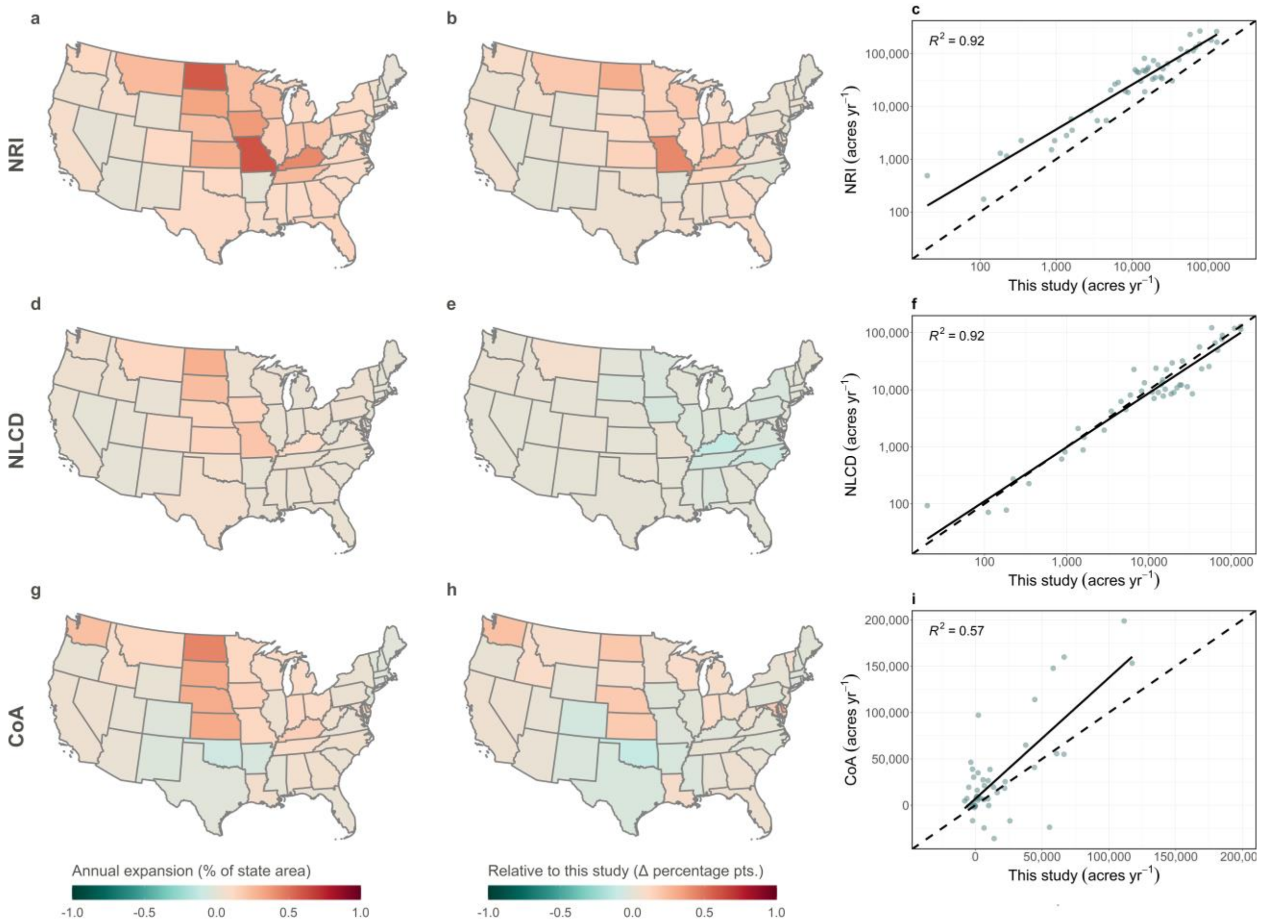

Supplementary Figure 14: State-level comparison to other recent cropland expansion estimates. Average annual rates of cropland expansion derived from the National Resources Inventory (NRI) 2007-15 (**a-c**), the National Land Cover Database (NLCD) 2008-16 (**d-f**), and the Census of Agriculture (CoA) 2007-17 (**g-i**). Maps in the first column (**a**, **d**, and **g**) depict the annual rate at which croplands expanded within each state, calculated as a percentage of the total state area. Maps in the second column (**b**, **e**, and **h**) show how these annual rates differ in absolute terms (Δ percentage points) from the comparable estimates derived in this study. Scatterplots in the third column (**c**, **f**, and **i**) illustrate how absolute annual rates of expansion within each state (acres yr^{-1}) from each data source compare to those in this study. The R^2 value for each linear regression is listed on the corresponding plot, with full regression statistics reported in **Supplementary Table 6**. Note that NRI and NLCD estimates and comparisons to this study are based on rates of gross cropland expansion; CoA estimates and comparisons based on rates of net cropland change. All three comparison datasets – based on unique sources – corroborate the general trend observed in this study of widespread cropland expansion throughout the US over approximately the past decade.

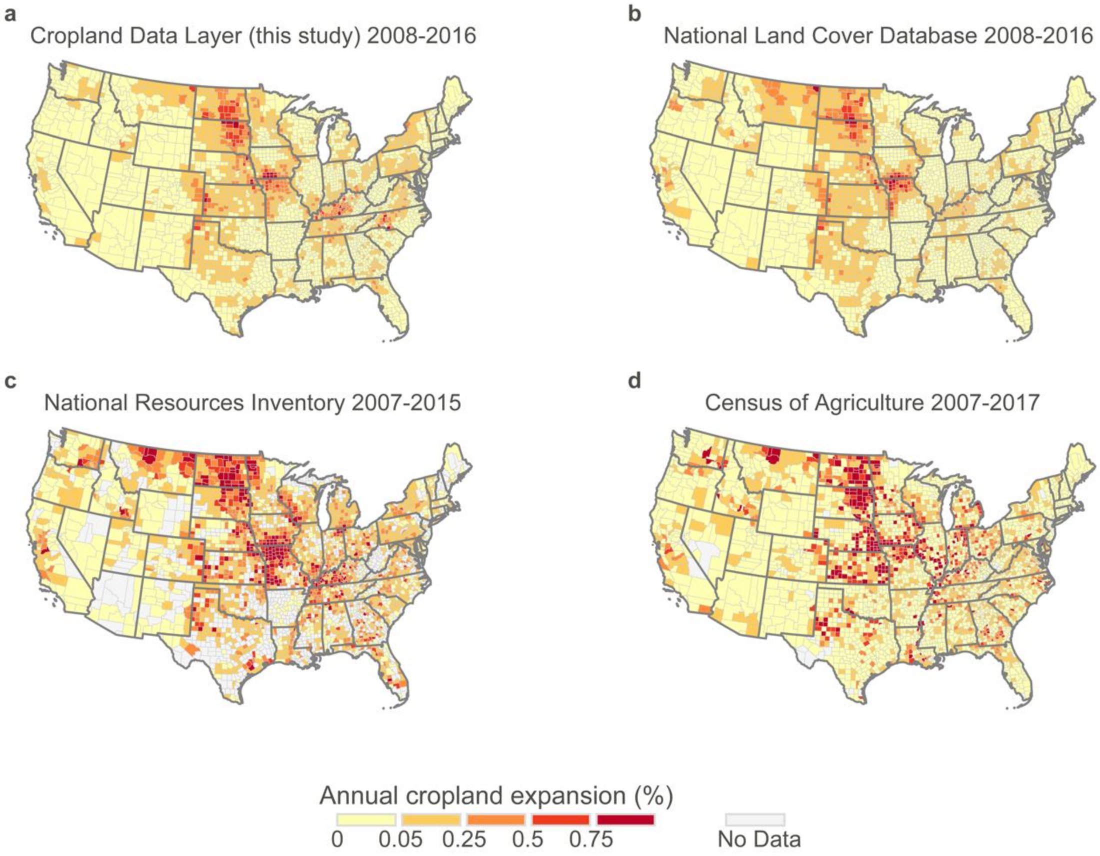

Supplementary Figure 15: County-level visual comparison of recent cropland expansion estimates. Maps depict (**a**) gross conversion from noncropland to cropland 2008-16 from this study; (**b**) gross conversion to cultivated cropland 2008-16 according the National Land Cover Database (NLCD); (**c**) gross transitions to cultivated and noncultivated cropland 2007-15 according to the National Resources Inventory (NRI); and (**d**) net increases in cropland area 2007-17 according to the Census of Agriculture (CoA). Reported values are the annual averages across each study period and reflect the amount of land that was converted within each county as a percentage of total county land area. Grey-colored counties represent administrative units with no data due to the sample size (NRI) or suppression for confidentiality (CoA). Despite their different data sources and analyses, the four mapped estimates indicate similar patterns of extensive cropland expansion over approximately the past decade in the US.

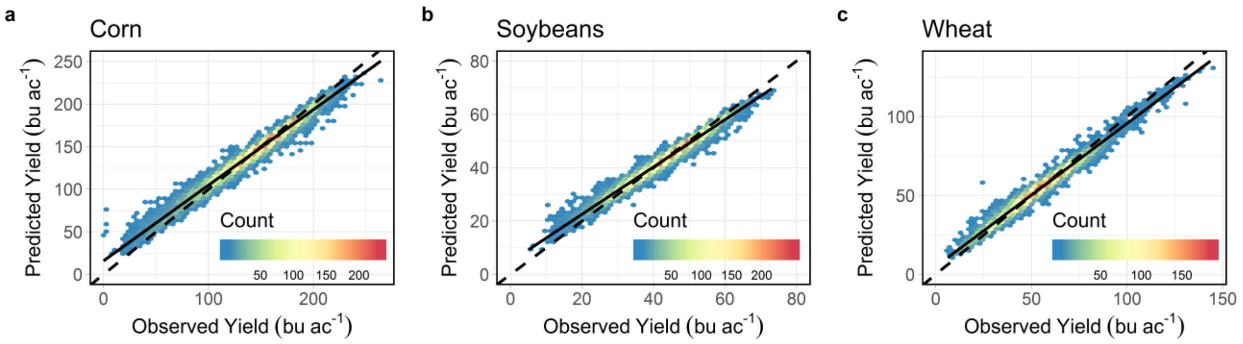

Supplementary Figure 16: Model validation results based on comparison between the predicted and observed yields for all cropland within a county for crop years 2008-17. Observed yield data are from the county-level NASS annual surveys. Model RMSE was 7.23, 1.97, and 3.30 bu ac^{-1} for corn, soybeans, and wheat, respectively.

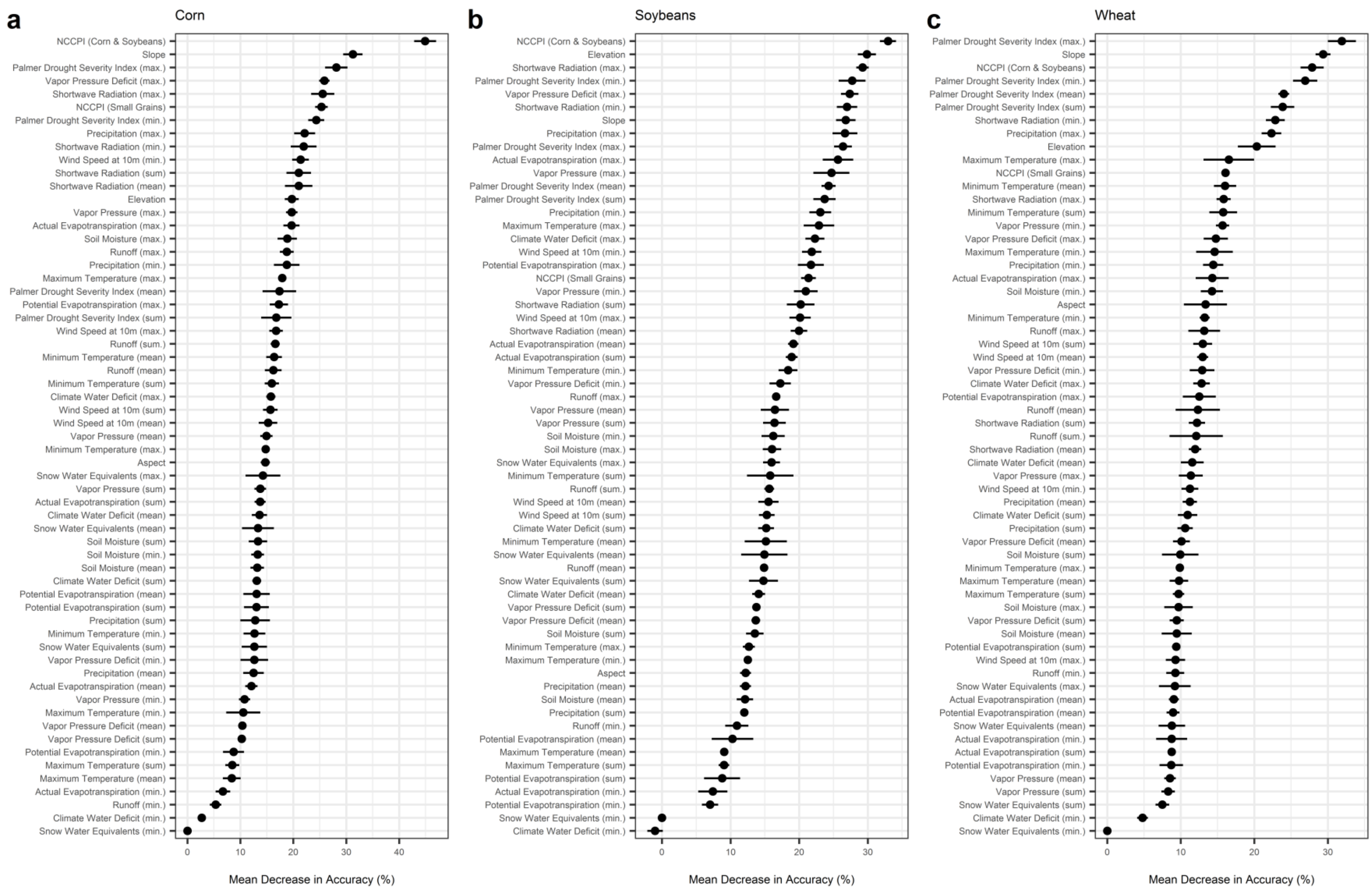

Supplementary Figure 17: Variable importance plots for the representative yield models of (a) corn, (b) soybeans, and (c) wheat. Mean decrease in accuracy reflects the decline in out-of-bag performance if a given variable is removed from the model. Variables with greater importance are located near the top of the plots. Whiskers represent the standard deviations of a 10-fold cross validation.

Supplementary Tables

Supplementary Table 1: Gross cropland expansion and abandonment, by state, 2008-16.

State	Expansion (acres)	Abandonment (acres)	Net Conversion % of state	Net Conversion acres	Net Conversion area
**Alabama**	160,700	51,400	0.33%	109,300	
**Arizona**	37,100	25,300	0.02%	11,800	
**Arkansas**	27,700	44,100	-0.05%	-16,400	
**California**	153,300	170,500	-0.02%	-17,200	
**Colorado**	329,000	121,800	0.31%	207,200	
**Connecticut**	1,800	3,600	-0.06%	-1,800	
**Delaware**	1,500	15,100	-1.05%	-13,600	
**Florida**	87,900	138,100	-0.14%	-50,200	
**Georgia**	112,400	58,100	0.14%	54,300	
**Idaho**	97,700	125,000	-0.05%	-27,300	
**Illinois**	175,600	43,300	0.37%	132,300	
**Indiana**	92,400	47,800	0.19%	44,600	
**Iowa**	548,800	61,500	1.35%	487,300	
**Kansas**	615,500	83,500	1.01%	532,000	
**Kentucky**	430,200	75,800	1.37%	354,400	
**Louisiana**	47,600	86,800	-0.13%	-39,200	
**Maine**	7,700	14,000	-0.03%	-6,300	
**Maryland**	22,800	86,800	-0.95%	-64,000	
**Massachusetts**	2,800	3,300	-0.01%	-500	
**Michigan**	119,800	31,900	0.24%	87,900	
**Minnesota**	349,800	47,500	0.56%	302,300	
**Mississippi**	104,200	23,700	0.26%	80,500	
**Missouri**	624,800	93,600	1.19%	531,200	
**Montana**	463,200	106,900	0.38%	356,300	
**Nebraska**	510,800	43,500	0.94%	467,300	
**Nevada**	11,000	21,000	-0.01%	-10,000	
**New Hampshire**	900	2,100	-0.02%	-1,200	
**New Jersey**	7,000	19,800	-0.26%	-12,800	
**New Mexico**	117,600	64,900	0.07%	52,700	
**New York**	198,700	167,900	0.10%	30,800	
**North Carolina**	270,300	94,800	0.55%	175,500	
**North Dakota**	1,033,100	141,500	1.97%	891,600	
**Ohio**	126,200	49,600	0.29%	76,600	
**Oklahoma**	202,900	91,400	0.25%	111,500	
**Oregon**	52,700	44,200	0.01%	8,500	
**Pennsylvania**	190,100	114,200	0.26%	75,900	
**Rhode Island**	200	2,200	-0.28%	-2,000	
**South Carolina**	42,000	27,100	0.07%	14,900	
**South Dakota**	1,044,400	105,000	1.90%	939,400	
**Tennessee**	234,700	56,600	0.66%	178,100	
**Texas**	881,600	436,200	0.26%	445,400	
**Utah**	65,300	54,300	0.02%	11,000	
**Vermont**	13,000	52,500	-0.64%	-39,500	
**Virginia**	149,300	98,300	0.20%	51,000	
**Washington**	130,700	112,200	0.04%	18,500	
**West Virginia**	13,000	9,100	0.03%	3,900	
**Wisconsin**	116,000	99,800	0.05%	16,200	
**Wyoming**	70,700	51,700	0.03%	19,000	
**United States**	**10,096,300**	**3,519,400**	**0.34%**	**6,576,900**	

Supplementary Table 2: Expected yields on new croplands relative to existing croplands nationwide.

Crop	Proportion of new cropland area (%)	Mean yield difference (%)	Standard Deviation (spatial)	Proportion of new cropland area with negative differential (%)
Corn	29.3	-10.9	13.8	78
Soy	26.7	-8.4	14.9	69
Wheat	22.6	1.3	21.1	59
Total*	78.6	-6.5	-	69.5

*Total represents the area-weighted average for "Mean yield difference" and "Proportion of new cropland area with negative differential"

Supplementary Table 3: Results from quantile regression analyses of the national and local yield differentials (%) in relation to the amount of natural cover remaining (%). Negative slopes indicate a lower absolute differential (smaller positive or greater negative value) within a 10 km x 10 km grid cell as the percentage of natural land cover increased within a grid cell. For example, a slope of -0.21 means that for every one percent increase in the amount of remaining natural land within a 10 x 10 km pixel, the yield difference decreased by 0.21%.

	National Yield Differential							Local Yield Differential						
		Slope			Intercept				Slope			Intercept		
**Crop**	**N**	**Estimate (s.e.m.)**	***t***	***p***	**Estimate (s.e.m.)**	***t***	***p***	**N**	**Estimate (s.e.m.)**	***t***	***p***	**Estimate (s.e.m.)**	***t***	***p***
Corn	32980	-0.211 (0.00285)	-74.2	0.00000	1.53 (0.197)	7.81	0.00000	31134	0.0106 (0.00047)	22.6	0.00000	-1.47 (0.0313)	-47.0	0.00000
Soy	26425	-0.208 (0.00392)	-53.1	0.00000	5.36 (0.285)	18.8	0.00000	25088	0.00466 (0.00026)	18.2	0.00000	-0.584 (0.0172)	-34.0	0.00000
Wheat	32337	-0.150 (0.00869)	-16.8	0.00000	28.1 (0.604)	46.5	0.00000	30570	0.00269 (0.00038)	7.0	0.00000	-0.411 (0.0243)	-17.0	0.00000

Supplementary Table 4: Expected yields on new croplands relative to existing local croplands within a 10 km x 10 km neighborhood.

Crop	Proportion of new cropland area (%)	Mean yield difference (%)	Standard Deviation (spatial)	Proportion of new cropland area with negative differential (%)
Corn	29.3	-1.1	1.8	78
Soy	26.7	-0.6	1.1	75
Wheat	22.6	-0.7	2.9	55
Total*	78.6	-0.8	-	70.4

*Total reflects the area-weighted average for "Mean yield difference" and "Proportion of new cropland area with negative differential"

Supplementary Table 5: Cultivation suitability of all land use classes according to the NRCS Land Capability Classification (LCC) system. LCC 1 is the most suitable for cultivation (prime), whereas LCC 8 is considered the least suitable for cultivation. The proportion of each land use class categorized as each LCC, 1-8, is displayed in each cell. In general, new croplands (i.e. cropland expansion) were less suitable for cultivation than current cropland extent (i.e. stable cropland) — the mean LCC of new croplands was 3.27, whereas the mean of existing crop fields was just 2.80, indicating greater limitations for new cropland.

	Proportion of each land use class categorized as LCC suitability 1-8								
**Broad land use class**	**1**	**2**	**3**	**4**	**5**	**6**	**7**	**8**	**Average LCC**
Stable Noncropland	0.6%	10.6%	13.8%	12.3%	2.2%	23.3%	33.5%	3.8%	5.28
Stable Cropland	5.9%	46.4%	28.4%	10.9%	0.9%	4.8%	2.6%	0.1%	2.80
Cropland Expansion	1.2%	32.6%	34.7%	17.9%	1.0%	8.9%	3.6%	0.2%	3.27
Cropland Abandonment	2.4%	31.2%	31.4%	16.4%	1.7%	9.2%	7.5%	0.3%	3.43
Intermittent Cropland	1.8%	27.7%	31.3%	17.5%	2.0%	11.1%	8.1%	0.5%	3.58

Supplementary Table 6: Linear regression statistics from comparisons to other datasets. Table values correspond to the results of the regression analyses performed at the levels of agricultural districts **(Figure 7)** and U.S. states **(Supplementary Figure 14)**. Data for analyses with the NRI and NLCD were log-log transformed to conform with the assumptions of ordinary least squares regression. In such cases, the equation takes the form log(y + 1) = slope*log(x+1) + intercept. For the untransformed CoA analysis, the equation takes the form y = slope*x + intercept. $RMSE_{CV}$ is calculated as the RMSE divided by the corresponding mean value of the points along the x axis in the figures (i.e. acres yr^{-1}, this study).

Comparison Dataset	N	Slope (SE)	Intercept (SE)	R^2	$RMSE_{CV}$
Enumeration unit: USDA Agricultural Districts					
NRI*	301	0.743 (0.0325)	2.86 (0.249)	0.636	9.85%
NLCD*	303	0.917 (0.0285)	0.450 (0.218)	0.775	10.0%
CoA	303	1.28 (0.130)	1190 (760)	0.243	251%
Enumeration unit: U.S. States					
NRI*	48	0.851 (0.0372)	2.33 (0.346)	0.919	4.85%
NLCD*	48	0.948 (0.0416)	0.348 (0.388)	0.919	6.05%
CoA	48	1.31 (0.167)	7020 (5580)	0.571	111%

*represents relationships that were log-log transformed

Supplementary Table 7: Comparison with other estimates of cropland expansion. The four national products compared here — the NLCD, the CoA, the NRI, and our study — all report annual average rates of net cropland expansion between 822,000 and 1.39 million acres, which represents a consensus of net cropland expansion during the last decade in the U.S.

Product	Comparable term(s)	Years reported	Total change (acres)	Average change per year (acres)
National Land Cover Database (NLCD)	Cultivated Crops (class 82)	2008-16	Exp: 9,682,841<br>Abn: 1,777,646<br>Net: 7,905,195	Exp: 1,210,355<br>Abn: 222,206<br>**Net: 988,149**
CDL-based (this study)	Cropland (see supp. methods)	2008-16	Exp: 10,096,300<br>Abn: 3,519,400<br>Net: 6,576,900	Exp: 1,262,000<br>Abn: 439,900<br>**Net: 822,100**
USDA Census of Agriculture (CoA)	Harvested + Failed + Fallow	2007-17	Exp: n/a<br>Abn: n/a<br>Net: 13,919,458	Exp: n/a<br>Abn: n/a<br>**Net: 1,391,946**
National Resources Inventory (NRI)	Cropland (Cultivated and Noncultivated)	2007-15	Exp: 21,458,200<br>Abn: 13,940,300<br>Net: 7,517,900	Exp: 2,682,275<br>Abn: 1,742,538<br>**Net: 939,738**

Supplementary Table 8: Expected accuracies of conversion classes across the study period. Expected accuracies calculated as the product of the state- and class-specific superclass accuracies for the year and class preceding and following conversion for each converted pixel. Nationwide, expected user's and producer's accuracies for conversion from noncropland to cropland were 71.0% and 86.9% on average across the study period, respectively, and ranged from lows of 62.2% and 83.3% in 2010 to highs of 92.6% and 94.3% in 2016. Average expected user's and producer's accuracies for abandonment during the study were 72.6% and 81.4%, respectively.

	Expansion		Abandonment	
**Year**	**User's**	**Producer's**	**User's**	**Producer's**
2009	63.3%	84.3%	67.0%	80.7%
2010	62.2%	83.3%	74.6%	84.0%
2011	71.4%	86.7%	61.9%	80.5%
2012	68.7%	87.3%	75.0%	78.6%
2013	81.2%	88.4%	74.0%	76.3%
2014	72.2%	88.3%	80.1%	76.5%
2015	73.0%	88.4%	84.0%	80.2%
2016	92.6%	94.3%	88.4%	84.5%
**Weighted Ave.**	**71.0%**	**86.9%**	**72.6%**	**81.4%**

Supplementary Table 9: Cropland and noncropland categorization of classes within the Cropland Data Layer. Each original class of the CDL was associated with a broad cropland or noncropland category for use in spatial processing and detection of change.

ID	Crop	ID	Crop	ID	Crop	ID	Non-Crop
1	Corn	48	Watermelons	216	Peppers	37	Other Hay/Non Alfalfa
2	Cotton	49	Onions	217	Pomegranates	62	Pasture/Grass
3	Rice	50	Cucumbers	218	Nectarines	63	Forest
4	Sorghum	51	Chick Peas	219	Greens	64	Shrubland
5	Soybeans	52	Lentils	220	Plums	65	Barren
6	Sunflower	53	Peas	221	Strawberries	81	Clouds/No Data
10	Peanuts	54	Tomatoes	222	Squash	82	Developed
11	Tobacco	55	Caneberries	223	Apricots	83	Water
12	Sweet Corn	56	Hops	224	Vetch	87	Wetlands
13	Pop or Orn Corn	57	Herbs	225	Dbl Crop WinWht/Corn	88	Nonag/Undefined
14	Mint	58	Clover/Wildflowers	226	Dbl Crop Oats/Corn	92	Aquaculture
21	Barley	59	Sod/Grass Seed	227	Lettuce	111	Open Water
22	Durum Wheat	60	Switchgrass	229	Pumpkins	112	Perennial Ice/Snow
23	Spring Wheat	61	Fallow/Idle Cropland	230	Dbl Crop Lettuce/Durum Wht	121	Developed/Open Space
24	Winter Wheat	66	Cherries	231	Dbl Crop Lettuce/Cantaloupe	122	Developed/Low Intensity
25	Other Small Grains	67	Peaches	232	Dbl Crop Lettuce/Cotton	123	Developed/Med Intensity
26	Dbl Crop WinWht/Soy	68	Apples	233	Dbl Crop Lettuce/Barley	124	Developed/High Intensity
27	Rye	69	Grapes	234	Dbl Crop Durum Wht/Sorghum	131	Barren
28	Oats	70	Christmas Trees	235	Dbl Crop Barley/Sorghum	141	Deciduous Forest
29	Millet	71	Other Tree Crops	236	Dbl Crop WinWht/Sorghum	142	Evergreen Forest
30	Speltz	72	Citrus	237	Dbl Crop Barley/Corn	143	Mixed Forest
31	Canola	74	Pecans	238	Dbl Crop WinWht/Cotton	152	Shrubland
32	Flaxseed	75	Almonds	239	Dbl Crop Soybeans/Cotton	171	Grassland Herbaceous
33	Safflower	76	Walnuts	240	Dbl Crop Soybeans/Oats	181	Pasture/Hay
34	Rape Seed	77	Pears	241	Dbl Crop Corn/Soybeans	176	Grassland/Pasture
35	Mustard	204	Pistachios	242	Blueberries	190	Woody Wetlands
36	Alfalfa	205	Triticale	243	Cabbage	195	Herbaceous Wetlands
38	Camelina	206	Carrots	244	Cauliflower		
39	Buckwheat	207	Asparagus	245	Celery		
41	Sugarbeets	208	Garlic	246	Radishes		
42	Dry Beans	209	Cantaloupes	247	Turnips		
43	Potatoes	210	Prunes	248	Eggplants		
44	Other Crops	211	Olives	249	Gourds		
45	Sugarcane	212	Oranges	250	Cranberries		
46	Sweet Potatoes	213	Honeydew Melons	254	Dbl Crop Barley/Soybeans		
47	Misc Vegs & Fruits	214	Broccoli				

Supplementary Table 10: Covariates considered by the random forest yield models. Gridded covariates were derived from the gSSURGO, TerraClimate, and USGS NED databases. Annual means of each covariate were tabulated within the planted extent of a given crop within each county in each year and joined to the corresponding county average yield of that crop in that year reported by the USDA's Agricultural Resource Management Survey. TerraClimate monthly grids were summarized as the multiyear average of the mean, total, minimum and maximum annual values between 2008-17, the period over which the yield model was implemented. TerraClimate grids had a native spatial resolution of 2.5 arc minutes and were resampled to 30m using the bilinear method prior to analysis. The gSSURGO and USGS NED layers are temporally static. The USGS NED had a native spatial resolution of 10m and was aggregated to 30m to match the land use change data, as well as the 30m resolution gSSURGO covariate grids.

Data Source	Covariate	Description
gSSURGO	NCCPIsg	National commodity crop productivity index for small grains
	NCCPIcs	National commodity crop productivity index for corn and soybeans
TerraClimate	aet	Actual evapotranspiration – derived using a one-dimensional soil water balance model. Mean, total, minimum and maximum annual (2008-17).
	def	Climate water deficit – derived using a one-dimensional soil water balance model. Mean, total, minimum and maximum annual (2008-17).
	pdsi	Palmer drought severity index – Mean, total, minimum and maximum annual (2008-17).
	pet	Reference evapotranspiration (ASCE Penman Monteith) – Mean, total, minimum and maximum annual (2008-17)
	pr	Precipitation accumulation – Mean, total, minimum and maximum annual (2008-17)
	ro	Runoff – derived using a one-dimensional soil water balance model. Mean, total, minimum and maximum annual (2008-17).
	soil	Soil moisture – derived using a one-dimensional soil water balance model. Mean, total, minimum and maximum annual (2008-17).
	srad	Downward surface shortwave radiation – Mean, total, minimum and maximum annual (2008-17).
	swe	Snow water equivalent – derived using a one-dimensional soil water balance model. Mean, total, minimum and maximum annual (2008-17).
	tmmn	Minimum temperature – Mean, total, minimum and maximum annual (2008-17).
	tmmx	Maximum temperature – Mean, total, minimum and maximum annual (2008-17).
	vap	Vapor pressure – Mean, total, minimum and maximum annual (2008-17).
	vpd	Vapor pressure deficit – Mean, total, minimum and maximum annual (2008-17).
	vs	Wind speed at 10m – Mean, total, minimum and maximum annual (2008-17).
USGS NED	Elevation	Elevation above sea level
	Slope	Terrain slope
	Aspect	Terrain aspect

Supplementary Note 1

Milkweed conversion

Relative impact and significance

The large number of milkweed stems lost due to land conversion reflects the substantial decline in CRP acreage over the past decade and the concomitant loss of pollinator habitat. Our estimate of 27.5 million milkweeds lost per year due to land conversion is over 14 times larger than the 1.9 million found by Pleasants (2016)[2], though our modeled extent comprises the broader 13-state region that encompasses the Pleasants modeled Midwest extent. On an area basis, our estimate of 53.7 stems lost per converted acre is roughly 11 times larger than the 4.9 stems per converted acre previously estimated[2]. This discrepancy arises primarily from a difference in accounting, in that the previous work included the loss of only non-CRP grasslands in its enumeration of land use change impacts (CRP was fully accounted for, but only in the estimate of milkweeds remaining, not milkweeds lost). By extending that analysis to consider the contribution of both CRP and non-CRP land conversion, we show both the precipitous decline of milkweed since 2008 as well as the significance of land use change relative to other drivers of milkweed loss. Most notably, the loss of milkweed due to recent land conversion is 26% as large as the mass extirpation of milkweed due to the adoption of glyphosate-tolerant crops and herbicide application that occurred between 1999 and 2014[2], which is estimated to be the single largest driver of recent Monarch population declines[3,4].

The loss of milkweed due to cropland expansion may also threaten Monarch recovery efforts, as milkweeds (*Asclepias* spp.) are the sole host plant for Monarch larvae[5]. Current Monarch conservation targets call for a doubling of existing milkweed populations, or the addition of another 1.3 billion stems. However, these recovery efforts will first need to overcome losses from ongoing crop expansion. If land conversion were to continue along the current composition and trajectory identified during our study period, additional restorations would be needed to make up for the 27.5 million stems lost each year in the Midwest before making progress towards the additional 1.3 billion stem goal. However, projected scenarios to achieve the restoration target already call for an “all hands on deck” approach to increase milkweed populations in every land sector just to meet the initial goal[5]. As such, there may be little room to increase restorations even further in order to compensate for the losses from conversion to cropland. The continued loss of habitat from land conversion should also be considered when updating future targets.

Estimating milkweed loss

Milkweed stems are commonly found in natural and managed grasslands, wetlands, and certain shrublands, but have been nearly extirpated from crop fields since the implementation of herbicide-tolerant crop varieties and associated pesticide application[5]. We estimated the total number of milkweed stems lost due to conversion of grasslands, shrublands, and wetlands to corn and soy production in the Midwest using the general approach of Pleasants (2016). For wetlands converted to crop production, we assumed an average initial stem density of 61.37 stems/acre[2].

For grasslands and shrublands converted to crop production, we first estimated the proportion likely to have been enrolled in the Conservation Reserve Program (CRP) prior to conversion using county-aggregated point data from the National Resources Inventory (NRI)[6]. Though county-level NRI data on the conversion of CRP to cropland were only available for 2008-15 at the time of analysis, we assume the same proportion of conversion from CRP extended throughout our study period of 2008-16.

Based on Thogmartin et al. (2017) we assumed a value of 3.09 stems/acre for grasslands and shrublands not enrolled in the Conservation Reserve Program and a density of 112.14 stems/acre for those lands enrolled in CRP. We then used the NRI-derived percentage of conversion that came from CRP in each county to estimate the acres of conversion from CRP and non-CRP and applied the associated stem densities. We also estimated average stems lost per acre based on the following equation:

$$\text{Stem density} = 3.09 * (1 - \text{CRP\%}) + 112.14 * (\text{CRP\%}) \qquad (1)$$

where "CRP%" is the proportion of new crop production that came from CRP within each county. Thus, in counties with no conversion of CRP to cropland, the stem density value used for all grasslands and shrublands converted to cropland was 3.09. In counties where nearly all conversion to cropland was from CRP, the value was near 112.14. For most counties, the stem density was an intermediate value, reflecting the mixed sources (CRP and non-CRP) of land converted to crop production.

We then used the same approach as above to estimate the existing 2008 stem populations based on the NRI percent of land enrolled in CRP in 2008 and 2008 land use from the CDL. To estimate relative losses in each county, we subsequently divided the number of stems lost by the number existing in 2008.

To enable equal comparison of total stems and average densities across states, we performed our analysis over the entire 13-state region encompassing the main summer breeding range for Monarchs in the Midwest[2], an area once estimated to support over 85% of the breeding population of monarch butterflies prior to the widespread loss of milkweed[5]. State level estimates of total stems lost, acres converted, and average stem density of converted lands are presented in **Supplementary Table 10**.

Supplementary Table 11: Number of milkweed stems lost 2008-16 due to conversion of grasslands, shrublands, and wetlands to corn and soybeans, by state.

State	Stems lost	Acres of conversion	Average stems lost per acre
Illinois	7,818,000	150,000	52.1
Indiana	1,621,000	82,000	19.8
Iowa	35,422,000	511,000	69.3
Kansas	12,065,000	250,000	48.3
Kentucky	7,689,000	384,000	20.0
Michigan	1,344,000	59,000	22.8
Minnesota	11,668,000	241,000	48.4
Missouri	25,874,000	569,000	45.5

Nebraska	23,969,000	430,000	55.7
North Dakota	41,723,000	492,000	84.8
Ohio	3,991,000	110,000	36.3
South Dakota	43,108,000	734,000	58.7
Wisconsin	4,037,000	92,000	43.9
13-State Region	220,330,000	4,103,000	53.7

Uncertainty and limitations in estimating milkweed loss

While our estimated milkweed loss numbers rely on the available data from the scientific literature, there remains substantial uncertainty in the magnitude of milkweed loss reported here. To help characterize this we calculated the standard errors for our estimates using reported error values for number of stems per acre from Pleasants (2016) and Thogmartin et al. (2017) for areas of wetlands, shrublands, CRP grasslands, and non-CRP grasslands based on the area of each from the estimates above[2,5]. In addition to that associated with the assumed stem densities, there also remain other sources of uncertainty that are not captured, as well as variation in the degree to which our data are representative. For example, many of the milkweed stem density values were based on observations in Midwestern states located at the interior of the modeled region, and thus values for milkweed stems and losses may be more uncertain and variable around the periphery of the region[5]. In addition, our estimates account for only common milkweed (*Asclepias syriaca*), and thus total and relative loss of all milkweed species may vary. Although recent field surveys suggest *syriaca* milkweeds outnumber the next most prevalent comparable variety by almost 10 to 1 in conservation grasslands in Minnesota, Wisconsin, and Iowa[7], other areas like Kansas and Missouri have higher occurrences of less common species like *Asclepias viridis,* and thus our estimates will be less representative of the changes occurring there[8]. Lastly, the estimates for milkweed stem densities are expected to vary widely from parcel to parcel and across landscape types, and thus the numbers presented here may represent overestimates in some areas and underestimates in others.

Supplementary Note 2

Comparison to other results

Comparison with other national data products

Our results are similar in direction but generally lesser in magnitude than other national assessments of cropland expansion. Compared to the National Land Cover Dataset (NLCD) we find nearly identical average annual rates of expansion, but approximately twice as much annual abandonment. The limited abandonment reported in the NLCD further exemplifies the difficulties in identifying abandonment from remote sensing data as described below. When compared to the National Resources Inventory (NRI), we report substantially lower levels of gross expansion and abandonment. This suggests the methodology of the NRI may be more sensitive to detecting conversion than that of either our CDL-derived or the NLCD satellite-based analyses. The USDA Census of Agriculture (CoA) reports the largest annual average rates of change of 1.39 million acres per year for the last 10 years. These farmer-reported data, often considered the gold standard of agricultural land use information[9], corroborate the independently assessed measurements and also suggest that producers are both aware of and acknowledge their active expansion of cultivated extent. Overall, the four national products compared here — the NRI, NLCD, CoA, and our study — report annual average rates of net cropland expansion of between 822,000 and 1.39 million acres, which represents a relative convergence and consensus on the extent of cropland area expansion during the last decade in the US.

Other common datasets used for comparison include national estimates from the United Nations' FAOSTAT database[10] and the USDA NASS annual surveys[11]. Data from the FAO regarding arable land and cropland extent for the US are based upon USDA CoA estimates for "total cropland" in the US. However, this broad USDA classification also includes subcategories of idle cropland and cropland-pasture, thereby cushioning its estimate of active cropland extent. Furthermore, there have been shifts within the definition and presentation of the cropland-pasture category of the CoA survey instrument over time[12]. These changes have led to discontinuity in land's classification as either cropland or pasture across time, thereby further muddling the use of USDA CoA estimates of total cropland and the associated FAOSTAT data points as indicators for active cropland area. Thus we made comparisons to only the specific categories in the USDA Census of Agriculture that best reflect active cropland extent — the sum of planted, failed, and fallow cropland — rather than the aggregated metric of total cropland reported by USDA and reflected by the FAO data.

Similarly, we did not compare results with those from NASS annual surveys of planted areas[11], as the annual survey data cannot be reliably used to estimate total cropland change due to multiple confounding factors. First, these surveys contain incomplete spatial coverage, and only report the planted area of specific crops for select counties in which they are economically important. For example, in 2017, corn planted acreage was reported for only 1635 of the 3070 counties across the US, despite corn being harvested in over 2600 counties according to the more comprehensive Census of Agriculture. Second, the annual NASS Surveys report only select principle crops rather than all crops, which thereby reduces the total area of cropped land enumerated. Third, the Surveys report only planted area, which is not an appropriate indicator of total cropland extent or the total footprint used

for cropland. To fully capture the footprint of cultivated agriculture (i.e. the area used for cropland), one would need to add to planted area the area of cropland that is annually tilled but not planted (i.e. fallow) as well as the areas of "prevented planting," which are croplands where farmers were unable to seed a crop and instead collected an insurance payment for their missed planting. The metrics of planted area and prevented planting are also heavily influenced by the weather and other local variables at the time of planting each year, and thereby fluctuate widely. Lastly, unlike the estimates based on the NRI, NLCD, and CDL, the NASS Survey planted area estimates provide only a measure of net area each year, rather than a spatially explicit tracking of land which would afford measurement of gross (to and from) land use changes.

Comparison to Lark et al. 2015

Compared to previous analyses of the CDL, we found 6.4 million acres of new cropland expansion during 2008-12, which is less than the 7.3 million acres of cropland expansion reported by Lark et al (2015) for the same period[13]. While some differences stem from updates to input data and methods (see **Supplementary Methods**), most is due to the longer period considered during classification in the current study. For example, land converted to crop production in 2008-12 and subsequently abandoned during 2012-16 would be captured in our intermittent cropland category rather than our crop expansion category, thus reducing the amount of conversion reported for 2008-12 in this study.

Notes about cropland abandonment

It remains a challenge to map and quantify recent cropland abandonment using remote-sensing derived land cover maps, due to multiple factors[14,15]. First, without knowing future, yet-to-be determined land use, it is not possible to distinguish short term fallowing or idling of land from longer term abandonment or removal from production, unless the destination use is a permanent land cover like urban development. Second, the time needed for growth and establishment of subsequent noncrop land covers like shrubland and forest similarly preclude estimating the amount of recent cropland that has been returned to these ecosystems in contemporary analyses. Lastly, the remaining bare ground and successive vegetative growth signals of newly abandoned croplands can appear spectrally similar to that of cropland, making it challenging to identify noncropland that immediately follows cropland when performing annual classifications. As such, future research that seeks to improve the characterization of recent abandonment (including grassland restoration mapping) might well focus on using linked time series analysis or continuous change detection methods as well as longer periods of analysis.

The overall area of abandonment we identified for 2008-16 is only slightly larger than that found for 2008-12 by Lark et al. 2015. In addition to lower rates of abandonment in recent years, much of the land identified as abandoned in the previous study was subsequently recultivated during the 2012-16 period and thus, more appropriately, was included in our pool of intermittent cropland.

Loss of cropland to urban expansion is difficult to measure since it frequently occurs over a period greater than one year, and during this process progresses through multiple land cover types. For example, we found large patches of orange groves in Florida that were cleared and subsequently left

vacant prior to development outside Fort Lauderdale and Miami. Other studies with an explicit focus on urban expansion may thus provide a better estimate of the rates of crop to urban conversion[6,16].

Additional challenges related to measuring abandonment may stem from the CDL data themselves. The CDL has increasingly captured more cropland over time, which, if uncorrected, leads to an overestimation of crop expansion and an underestimation of cropland abandonment. We adjusted for this bias toward overestimating expansion by using the NLCD to correct the few areas of potential expansion falsely identified by the CDL as noncropland in earlier years[17]. However, a corresponding approach to correct for missed cropland that had been subsequently abandoned does not exist as it would require application of the NLCD to all possible lands (rather than the small pool of potential conversion locations).

Supplementary Note 3

Yield model interpretation

Yield model predictions are best interpreted as mean expected yields 2008-17 based on the land's biophysical characteristics and a given county's dominant management practices. Each model integrates primarily static biophysical variables and their long-term relationship to observed yields. Since ten years of yield data were considered equally, predictions best represent the average value of the corresponding time period and do not account for potential improvements arising from subsequent genetic or management advances within the study period. Thus we report only relative yield patterns between new and existing croplands, which are less likely to be affected by these advances since they are adopted quickly and uniformly.

Supplementary Methods

Estimating conversion accuracy

We used the data reported in the CDL error supermatrices to estimate the accuracy of a conversion between noncropland and cropland. Initially, NASS calculates the accuracy for specific land cover classes within each state according to the general formula:

$$\text{Class accuracy}_x = \frac{\text{Pixels correct}_x}{\text{Pixels total}_x} \qquad (2)$$

for each specific crop *x*, where *pixels correct* is the number of mapped pixels that match the reference data in a given region, and *pixels total* is either the total number of reference data observations (for calculating producer's accuracy) or mapped pixels (for calculating user's accuracy) for each class. Producer's accuracies measure errors of omission; they indicate how likely a feature is to be correctly captured by the remote sensing product. User's accuracies reflect errors of commission, which indicate how likely a mapped class correctly resembles features on the landscape (Congalton and Green, 2008).

Aggregating land cover classes to broader thematic classes improves accuracy by lowering thematic specificity [20,21]. To understand how well the CDL data used as input can distinguish general cropland from noncropland areas, we calculated how frequently each *specific* class of the CDL is correctly mapped within the appropriate cropland or noncropland domain. This metric indicates, for example, how accurately a pixel mapped as corn can be used to identify general cropland. We refer to this as the superclass accuracy for each specific class, and derived it as:

$$\text{Superclass accuracy}_{C,x} = \frac{\text{Pixels in correct superclass}_x}{\text{Pixels assessed}_x} \qquad (3)$$

for each specific crop *x* included in the superclass or domain *C* (e.g., cropland or noncropland). The superclass producer's accuracy indicates how frequently a specific crop on the landscape, such as corn, was mapped by the CDL as any type of crop in the cropland domain. The superclass user's accuracy represents how likely a pixel mapped as a specific crop was actually any type of crop (i.e. cropland) on the landscape.

These superclass accuracies were then used to estimate the likelihood that a conversion was correct by multiplying the state- and class-specific superclass accuracy of each converted pixel for the specific year and class preceding and following conversion (eq 3).

$$\text{Expected Accuracy} = SA_{yoc} * SA_{(yoc-1)} \qquad (4)$$

where SA is the superclass accuracy and yoc is the year of conversion. This approach provides a thematically and temporally explicit estimate of the expected accuracy for each land use change identified.

Detecting land conversion

The land cover change detection process was divided into four sequential stages: 1) pre-processing, which consisted of compiling the original trajectories of land cover through time; 2) specific class refinements, which involved editing individual pixels and trajectories to account for known common misclassification issues; 3) core processing, which included spatiotemporal filtering, categorization of the unique landcover trajectories into five broad land use change classes, and application of a minimum mapping unit to the broad classes; and 4) post-processing, which consisted of identifying the years of conversion and the specific landcover classes before and after each conversion occurred (**Supplementary Fig. 16)**

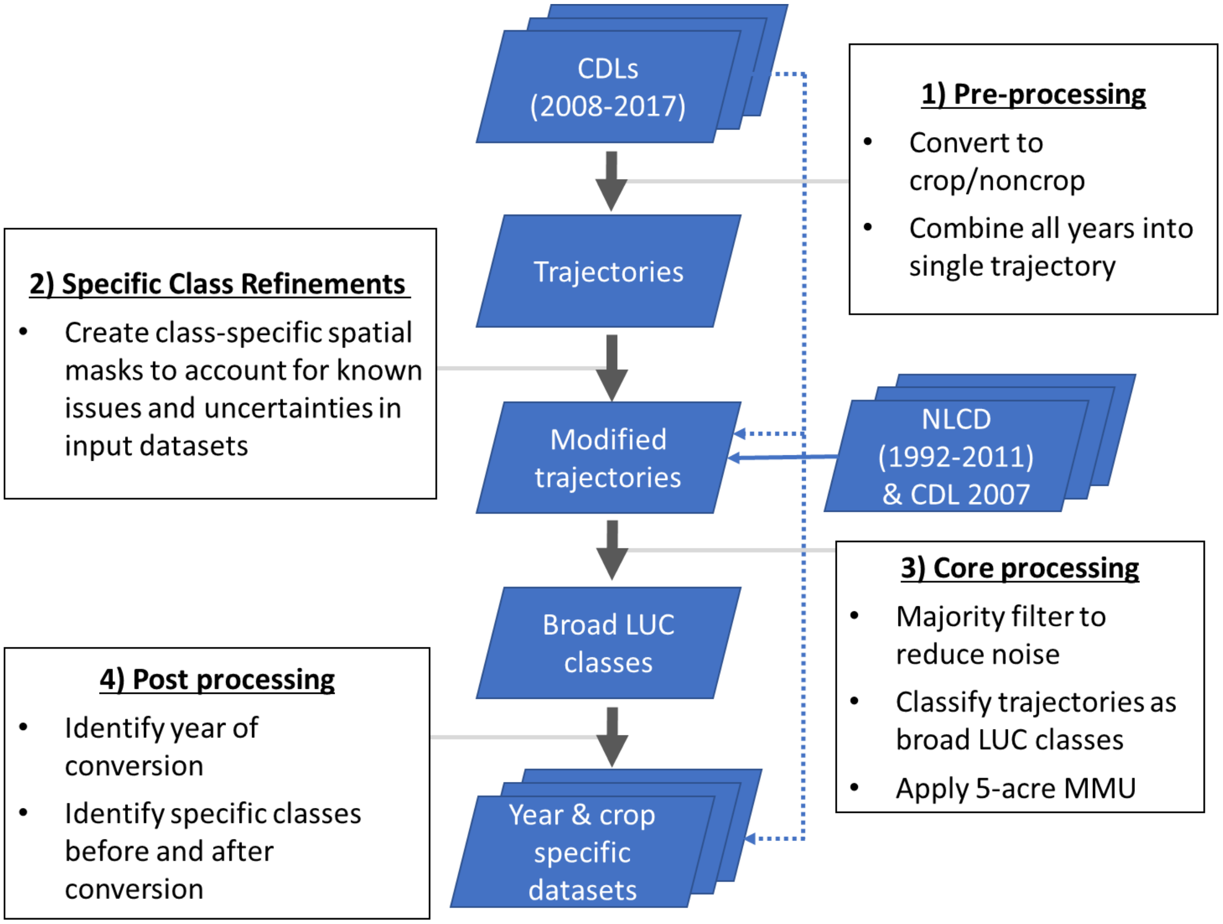

Supplementary Figure 18: Overview of the general workflow for detecting land conversion using the Cropland Data Layer and National Land Cover Database. Processing was broken down into four general stages: pre-processing, specific class refinements, core processing, and post-processing.

1) Pre-processing

We used the USDA Cropland Data Layer (CDL) as the primary input for detecting land conversion. The CDL is an annual 30m resolution, crop-specific land cover map that provides coverage for all states in the conterminous U.S. beginning in 2008, with crop-specific accuracies generally ranging from 85-95%[1]. As a first processing step, we reclassified all CDL datasets available at the time of analysis (2008-17) into a binary classification of either cropland or noncropland according to **Supplementary Table 8**. We then combined all ten reclassed CDL datasets to create a single 'trajectory' dataset containing 1024 (i.e. 2^10) unique values, one for each permutation of input values. These trajectories represented the unique combinations of land use across time.

2) Specific class refinements

Next, we used the original crop-specific CDLs to modify a selection of the 1024 binary land use trajectories to help account for known issues and uncertainties in the raw CDL input datasets. These modification masks were created by generating class-specific data layers based on the original CDLs and then spatially tagging them to the cropland/noncropland trajectories. Using this approach, we were able to leverage and maintain the thematic richness of the original CDLs (which often contain over 100 classes each year) while maintaining a tractable number of trajectories based on the annual binary cropland/noncropland maps. These refinement masks were applied to the trajectory dataset immediately after the pre-processing stage in order to maintain spatial alignment throughout the subsequent processing steps. All of the applied modification masks were designed to reduce errors via the removal of false positive signals (i.e. identification of conversion when it is likely that no conversion occurred) or via compensation for false negatives (i.e. failure to identify conversions that likely occurred).

Adjusting for potential missed abandonment

The fallow/idle cropland class of the CDL is intended to capture cultivated or tilled land that was not planted to a specific crop in a given year but still actively managed as cropland. This type of annual fallow land is often found in rotations with crops such as wheat, and commonly used as a water conservation practice in the western U.S. However, land identified as fallow can also indicate the start of an abandonment process if it is not followed by crop production in a subsequent year. Thus, by considering fallow/idle to be an active cropland class in our schema, some areas of conversion to noncropland (i.e. abandonment) could have been missed if a crop was followed by the fallow/idle class and never subsequently planted. To account for this, we reinspected all pixels of potential stable cropland or intermittent cropland to see whether an abandonment involving a fallow/idle classification may have occurred. Specifically, if an individual pixel i) contained only cropland classes in its initial years, ii) was subsequently classified as fallow, and iii) remained fallow or noncrop for the rest of the time series (i.e. unidirectional conversion), then the initial fallow/idle year was treated as the first noncropland year and the trajectory was labeled as abandonment. This corrective mask was applied to the trajectory dataset prior to the subsequent masks described below to allow those later corrections to supersede this refinement.

Adjusting for potential false expansion and false abandonment

We used additional rule-based processing to help remove areas identified as conversion (i.e. expansion or abandonment) that were likely to be misclassifications, based either on a low probability of that type of transition occuring or on low accuracy in distinguishing specific types of land cover involved in the change.

Pixels which would otherwise meet our criteria for a conversion (see below) but which contained a tree crop — specifically cherries (66), peaches (67), apples (68), grapes (69), other tree crops (71), citrus (72), pecans (74), almonds (75), walnuts (76), pears (77), pistachios (204), prunes (210), olives (211), oranges (212), nectarines (218), plums (220), or apricots (223) — in any year prior to and following the conversion year were relabeled as stable cropland due to the likelihood of that pixel being part of an orchard and the potential false conversion signal arising from the replanting or regrowth cycle. Similarly, any potential conversion pixel that contained rice (class 3) in any year prior to and following a potential conversion was relabeled as stable cropland due to the persistent nature of paddy agriculture.

Land that has been developed into urban or built-up infrastructure is unlikely to revert back to cultivated agriculture due to the substantial capital invested, the standing infrastructure, or soil degradation. Therefore, we did not allow developed land classes — specifically developed/low intensity (122), developed/medium intensity (123), and developed/high intensity (124) — to be considered as conversion to cropland under the assumption that any such signatures likely reflect a misclassification in the underlying data.

Alfalfa is the most frequently confused class in the cropland domain[18] and is often confused with nonalfalfa hay or grassland/pasture, both considered noncropland classes. Therefore, to prevent misclassified alfalfa pixels from inducing a false conversion signal, we required presence of an additional type of crop sometime after the conversion to alfalfa occurred.

Lastly, we created a broad class-specific filter to address pixels that contained low classification accuracy or that were classes that were frequently confused for the duration of the time series. If a pixel contained only landcover classes included in the designated fuzzy list — specifically alfalfa (36), other hay/non alfalfa (37), fallow idle cropland (61), shrubland (152), or grassland/pasture (176) — then it was removed as a conversion pixel because of the low confidence of conversion, low accuracy, and/or spectral similarity of landcover classes pre- and postconversion.

Incorporating the National Land Cover Dataset (NLCD).

We also used the independent NLCD dataset to help further refine conversion estimates by requiring agreement between the NLCD and the CDL-based trajectories. For areas of potential cropland expansion identified by the CDL, we required that the area was not classified as a cultivated crop (82) in either of the two previous NLCD datasets. For areas of potential cropland abandonment identified by the CDL, we required that the area was classified as either cultivated cropland (82) or pasture/hay (81) in at least one of the two previous NLCD datasets.

For potential abandonment, if there was no cultivated crop (82) or pasture/hay (81) in the two previous NLCD datasets, then we assumed that this pixel was not previously cropped consistently and therefore had no potential for abandonment. Such pixels were thus reclassified as noncropland.

Incorporating the 2007 Cropland Data Layer

The 2007 CDL dataset does not have full continental coverage and thus was excluded as one of the CDL inputs when creating the trajectories dataset. However, we leveraged these data for context wherever they were available. To use the 2007 CDL, the dataset was first reclassified to the binary cropland/noncropland scheme. This binary dataset was then referenced with all pixels preliminarily identified as potential conversion in 2009. For areas of potential cropland expansion, if the binary 2007 CDL was cropland then it disagreed with the potential cropland expansion pattern and the area was relabeled as cropland. Likewise, for areas of potential abandonment, if the binary 2007 CDL was noncropland then it disagreed with the potential abandonment pattern and the area was relabeled as noncropland.

3) Core processing

The core processing stage involved the creation of the broad land use change dataset and consisted of three steps, implemented in sequence: first, application of a spatial filter on the modified trajectories; next, categorization of unique landcover permutations into one of five broad land use transition classes; and finally, implementation of a minimum mapping unit to the broad LUC dataset.

Spatial filtering

The first step in the core processing sequence was to apply a spatial filter to the modified trajectories from the specific class refinement stage. The spatial filter was used to clean the dataset by reducing within-field salt-and-pepper potential misclassifications associated with CDL classifier confusion as well as potential edge-of-field confusion associated with mixed pixels. To determine which filter best modeled the landscape, we explored multiple parameters of two spatial filters. The first spatial filter we explored was the majority filter with number of neighbors and replacement threshold as the two parameters. The arguments for the number of neighbors parameter were four (orthogonal) and eight (a three-by-three window). Based on observations in several test regions, the four-neighbor option better retained original field shapes and reduced unnecessary smoothing at field corners.

The arguments for the replacement threshold for the majority filter were half versus majority thresholds. The half threshold more aggressively removed smaller features on the landscape than the majority threshold because of the lower threshold required for replacement. In general, we observed that these small patches were frequently composed of mixed pixels at the interface of crop and noncrop patches and often falsely identified conversions, and therefore it was desirable to remove them.

We also explored an alternative moving window filter with different kernel sizes (3 X 3) and (5 X 5) as the parameters. It was observed that larger kernel sizes created more homogenous patches, which resulted in a smoother modeled landscape with a reduction in spatial complexity and smaller features. These filters created fewer structurally complex patches and removed a larger number of smaller

patches relative to the majority filter because no minimum threshold was required for replacement. Although there were benefits to a more aggressive spatial filter that made patches more uniform in shape and composition, we chose the majority filter with eight neighbors and half threshold over the moving window filters because the output of the majority filter with chosen parameters preserved more heterogenous patches on the landscape and maintained a greater number of small features like conversion patches, small waterbodies (e.g., prairie potholes in North Dakota), roads, riparian areas, etc.

Broad Land Use Change (LUC) classification

The second step in the core processing sequence was to classify each of the unique trajectory values as one of five broad LUC categories. A trajectory was classified as cropland expansion if it contained a single sequence of two noncrop years followed by two crop years. Similarly, trajectories were classified as abandonment if they contained two crop years followed by two noncrop years within the study period. If the broad LUC categories were either expansion or abandonment, the year of the conversion was also attached to the record. If the trajectory sequence contained all cropland labels or all noncropland labels or contained just a single opposite crop/noncrop label, then the trajectory was classified as stable cropland or stable noncropland, respectively. All other trajectory permutations, including those which contained two or more conversions, were subsequently classified as intermittent cropland. Note that for 2009, an exception to the classification requirements for expansion and abandonment were required due to the lack of nationwide CDL coverage prior to 2008, and therefore we required only one year of preconversion cropland or noncropland CDL data. However, this impact was mitigated by later incorporating the 2007 CDL for all states in which it was available as well as requiring consistency with the two previous NLCD datasets (see step 2 refinements above).

Minimum Mapping Unit (MMU)

The third and final step in the core processing sequence involved applying an MMU of five acres to the broad LUC dataset. This MMU involved removing patches of broad LUC smaller than five acres and replacing them with the trajectories (and associated LUC classes) of the nearest pixel neighbors. Without replacement, the total area of land and each broad LUC class would be underestimated, and certain LUC types (e.g., small patches of conversion) could be systematically underrepresented. To perform the replacement, we filled the voided pixels using a nearest neighbor approach[19].

While testing various MMU parameters between zero and 15 acres, we found that larger MMU threshold sizes generally reduced the number of false conversions detected but increased the number of true conversions missed by the classification process. Based on observations using high resolution aerial imagery, we selected an MMU size of five acres to strike a balance between maintaining important features on the landscape (e.g., roads, broad LUC change categories, water bodies, riparian areas, hedgerows, highway rights-of-way) while still removing small patches of likely misclassification (e.g., edge-of-field mixed pixels and CDL misclassifications).

4) Post-processing

The fourth and final stage of LUC detection consisted of identifying the years of conversion and the specific landcover classes preceding and following conversion. In all, six datasets were created in this

stage — three for each conversion type (i.e., expansion and abandonment), which tracked the year of conversion, the land cover preceding a conversion, and the land cover immediately following conversion. To ensure consistency with the LUC product results, the attributes of these layers were derived from original CDL datasets that were modified with the same processing as above. For any areas of missing or mismatched data due to a replacement during spatial filtering, a final chained nearest neighbor operator with progressively larger neighborhoods was used to select the nearest valid land cover class.

Supplementary References

1. Boryan, C., Yang, Z., Mueller, R. & Craig, M. Monitoring US agriculture: the US Department of Agriculture, National Agricultural Statistics Service, Cropland Data Layer Program. *Geocarto Int.* **26**, 341–358 (2011).
2. Pleasants, J. Milkweed restoration in the Midwest for monarch butterfly recovery: estimates of milkweeds lost, milkweeds remaining and milkweeds that must be added to increase the monarch population. *Insect Conserv. Divers.* n/a-n/a (2016) doi:10.1111/icad.12198.
3. Stenoien, C. *et al.* Monarchs in decline: a collateral landscape-level effect of modern agriculture. *Insect Sci.* **25**, 528–541 (2018).
4. Thogmartin, W. E. *et al.* Monarch butterfly population decline in North America: identifying the threatening processes. *R. Soc. Open Sci.* **4**, 170760 (2017).
5. Thogmartin, W. E. *et al.* Restoring monarch butterfly habitat in the Midwestern US: 'all hands on deck'. *Environ. Res. Lett.* **12**, 074005 (2017).
6. USDA. *2015 National Resources Inventory: Summary Report*. http://www.nrcs.usda.gov/Internet/FSE_DOCUMENTS/nrcseprd396218.pdf (2018).
7. Lukens, L. *et al.* Monarch Habitat in Conservation Grasslands. *Front. Ecol. Evol.* **8**, (2020).
8. Orley, T. Personal communication. (2019).
9. Johnson, D. M. A 2010 map estimate of annually tilled cropland within the conterminous United States. *Agric. Syst.* **114**, 95–105 (2013).
10. FAOSTAT. http://faostat.fao.org/.
11. USDA - National Agricultural Statistics Service. *Guide to NASS Surveys* http://www.nass.usda.gov/Surveys/Guide_to_NASS_Surveys/index.php.
12. Bigelow, D. & Borchers, A. *Major uses of land in the United States, 2012*. (2017).
13. Lark, T. J., Salmon, J. M. & Gibbs, H. K. Cropland expansion outpaces agricultural and biofuel policies in the United States. *Environ. Res. Lett.* **10**, 044003 (2015).
14. Yin, H. *et al.* Mapping agricultural land abandonment from spatial and temporal segmentation of Landsat time series. *Remote Sens. Environ.* **210**, 12–24 (2018).
15. Estel, S. *et al.* Mapping farmland abandonment and recultivation across Europe using MODIS NDVI time series. *Remote Sens. Environ.* **163**, 312–325 (2015).
16. Theobald, D. M. & Dickson, B. G. Description of the approach, data, and analytical methods used for the Farms Under Threat: the State of America's Farmland project. (2018).
17. Lark, T. J., Mueller, R. M., Johnson, D. M. & Gibbs, H. K. Measuring land-use and land-cover change using the U.S. department of agriculture's cropland data layer: Cautions and recommendations. *Int. J. Appl. Earth Obs. Geoinformation* **62**, 224–235 (2017).
18. Lark, T. J. *America's Food- and Fuel-Scapes: Quantifying Agricultural Land-Use Change Across the United States*. (The University of Wisconsin - Madison, 2017).
19. Overmars, K. P., de Koning, G. H. J. & Veldkamp, A. Spatial autocorrelation in multi-scale land use models. *Ecol. Model.* **164**, 257–270 (2003).
20. Anderson, J. R. *A Land Use and Land Cover Classification System for Use with Remote Sensor Data*. (U.S. Government Printing Office, 1976).
21. Wickham, J. D., Stehman, S. V., Fry, J. A., Smith, J. H. & Homer, C. G. Thematic accuracy of the NLCD 2001 land cover for the conterminous United States. *Remote Sens. Environ.* **114**, 1286–1296 (2010).
